# The pathogenicity island encoded PvrSR/RcsCB regulatory network controls biofilm formation and dispersal in Pseudomonas aeruginosa PA14

**DOI:** 10.1111/mmi.12287

**Published:** 2013-06-28

**Authors:** Helga Mikkelsen, Kailyn Hui, Nicolas Barraud, Alain Filloux

**Affiliations:** 1 Imperial College LondonDepartment of Life SciencesMRC Centre for Molecular Bacteriology and InfectionSouth Kensington CampusFlowers Building, SW7 2AZ, London, UK; 2 School of Biotechnology and Biomolecular Sciences and Centre for Marine Bio-Innovation, University of New South WalesSydney, NSW, 2052, Australia

## Abstract

*Pseudomonas aeruginosa* biofilm formation is linked to persistent infections in humans. Biofilm formation is facilitated by extracellular appendages, some of which are assembled by the Chaperone Usher Pathway (Cup). The *cupD* gene cluster is located on the PAPI-1 pathogenicity island of strain PA14 and has probably been acquired together with four genes encoding two-component signal transduction proteins. We have previously showed that the RcsB response regulator activates expression of the *cupD* genes, which leads to the production of CupD fimbriae and increased attachment. Here we show that RcsB activity is tightly modulated by two sensors, RcsC and PvrS. While PvrS acts as a kinase that enhances RcsB activity, RcsC has a dual function, first as a phosphorelay, and second as a phosphatase. We found that, under certain growth conditions, overexpression of RcsB readily induces biofilm dispersal. Microarray analysis shows that RcsB positively controls expression of *pvrR* that encodes the phosphodiesterase required for this dispersal process. Finally, in addition to the PAPI-1 encoded *cupD* genes, RcsB controls several genes on the core genome, some of which encode orphan response regulators. We thus discovered that RcsB is central to a large regulatory network that fine-tunes the switch between biofilm formation and dispersal.

## Introduction

*Pseudomonas aeruginosa* is an environmental bacterium that thrives in diverse ecological niches including soil and water. It is also an opportunistic pathogen with a broad host range that causes a variety of nosocomial infections in humans, which are notoriously difficult to treat (Kulasekara and Lory, 25). One of the challenges of resolving *P. aeruginosa* infections is because of its ability to form biofilms that are highly resistant to the action of the immune system, as well as to antibiotic intervention (Harmsen *et al*., 14; Breidenstein *et al*., 2). Among the extracellular appendages that contribute to biofilm formation are fimbriae produced by the Chaperone Usher Pathway (Cup) (Vallet *et al*., 49; Waksman and Hultgren, 51). Work by us and others has shown that *P. aeruginosa* PAO1 has four different types of Cup fimbriae (CupA, CupB, CupC and CupE), whereas strain PA14 has an additional type, CupD, which is encoded on the horizontally acquired PAPI-1 pathogenicity island (Vallet *et al*., 49; Ruer *et al*., 42; Mikkelsen *et al*., 31; Giraud and de Bentzmann, 11). Although their specific biological function is unknown, all these fimbriae have been shown to contribute to biofilm formation (Vallet *et al*., 48; Kulasekara *et al*., 26; Mikkelsen *et al*., 31; Giraud *et al*., 10). Mutants in the *cupD* gene cluster are also attenuated in plant and animal models of infection (He *et al*., 15).

*Pseudomonas aeruginosa* has a large genome (over 6 Mb for most strains), and a high proportion of its coding capacity is dedicated to regulation (Stover *et al*., 44). Two-component systems (TCSs), which are the predominant signalling systems in most bacteria, are also well represented with well over 100 genes in strain PAO1 (Rodrigue *et al*., 40; Whitworth, 54). These systems continuously probe the environment and allow the bacteria to modify their behaviour accordingly. Many TCSs also control factors involved in multicellular behaviour, such as biofilm formation (Mikkelsen *et al*., 32). The majority of TCSs consists of a classical sensor kinase containing a cytoplasmic transmitter domain that autophosphorylates on a conserved histidine upon the detection of an input signal. The phosphoryl group is then transferred to a response regulator containing a conserved aspartate in the N-terminal receiver domain, which in turn activates the output domain, which is often a transcription factor (Galperin, 9). While these conventional TCSs are characterized by a single His-Asp phosphotransfer, phosphorelays enable multiple transfer events. Hybrid and unorthodox sensors thus have a receiver domain fused to the C-terminus and require a histidine phosphotransfer domain (Hpt), also known as a phosphorelay, to phosphorylate the response regulator. In the unorthodox sensor, this Hpt is an integral part of the protein. Activation of the response regulator in these atypical systems is therefore the result of an alternating His-Asp phosphorylation cascade. These arrangements are likely to give more flexibility and better fine-tuning of the regulation (Mikkelsen *et al*., 32; Whitworth, 54).

The hybrid and unorthodox sensors often participate in more complex regulatory systems that can involve either more than one phospho-donor or multiple phospho-acceptor proteins. For example, the phosphorylation state of LuxO in *Vibrio harveyi* is influenced by three sensor kinases, LuxN, CqsS and LuxQ (Waters and Bassler, 52). Conversely, in the Roc system of *P. aeruginosa*, the RocS1 and RocS2 sensors can both act through at least three different response regulators (RocA1, RocA2 and RocR), thereby forming a regulatory network (Kulasekara *et al*., 26; Sivaneson *et al*., 43; Qaisar *et al*., 38). Another example of an unconventional system is the Gac/Rsm system of *P. aeruginosa*, in which phosphorylation of the GacA response regulator by the unorthodox GacS sensor is influenced by at least two additional hybrid sensors, RetS and LadS (Ventre *et al*., 50; Goodman *et al*., 13).

We have previously shown that expression of the *cupD* gene cluster is inversely controlled by two response regulators. RcsB, which is predicted to bind DNA, activates expression of the *cupD* genes, while PvrR, which has an EAL motif and has been shown to degrade c-di-GMP (Meissner *et al*., 30), has a negative effect on *cupD* gene expression (Mikkelsen *et al*., 31; Nicastro *et al*., 36). In this study, we elucidate the role of the two associated sensor kinases, RcsC and PvrS, and show that they both act on the RcsB response regulator, but play very different roles. We also show that when grown in dynamic conditions such as in a microfermentor, the overexpression of RcsB induces biofilm dispersal. This dispersal process appears to entirely rely on the activity of the phosphodiesterase PvrR. We finally demonstrate that, in addition to the *cupD* gene cluster on the PAPI-1 pathogenicity island, the RcsB response regulator controls the expression of other regulators on the core genome. Although their specific role is unknown, these findings highlight the key role of the horizontally acquired RcsBC/PvrRS TCSs in modulating more globally the bacterial cell physiology.

## Results

### RcsC interacts with RcsB

We have previously shown that the *cupD* gene cluster is inversely regulated by the response regulators RcsB and PvrR (Mikkelsen *et al*., 31). The gene cluster encoding these regulators also encodes two putative sensor kinases. The hybrid sensor PvrS is encoded upstream of PvrR, and the unorthodox sensor RcsC is encoded upstream of RcsB. It is predicted that these four genes are in an operon (Mao *et al*., 29), suggesting that they belong to the same regulatory system. We therefore hypothesized that the regulation of the *cupD* gene cluster consisted of an activating pathway, RcsC–RcsB, and a repressing pathway, PvrS–PvrR. Since PvrS is a hybrid sensor, its potential phosphorylation of PvrR may also be expected to require an external Hpt module (Mikkelsen *et al*., 31).

**Figure 1:**
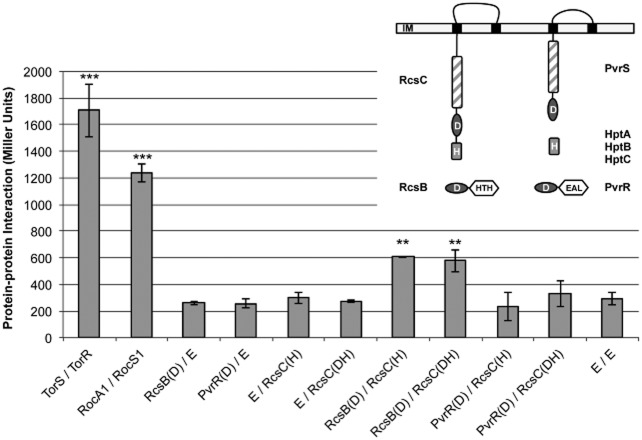
Protein–protein interaction between selected components of the Rcs/Pvr regulatory system investigated by bacterial two-hybrid analysis. Plasmids (pUT18c or pKT25 respectively) expressing relevant domains of potentially interacting proteins were co-transformed into *E. coli* DHM1 cells, which were grown on MacConkey agar. Interactions were quantified by β-galactosidase assays in biological triplicates. The TorR/TorS and RocA1/RocS1 two-component systems were included as positive controls. D indicates receiver domain, H indicates Hpt domain and DH indicates both. E indicates empty vector. Significant increases compared with the double empty vector control are indicated with asterisks (Student's *t*-test, **P ≤ 0.01; ***P ≤ 0.001). Inset: schematic diagram of proteins of interest with sensors inserted into the inner membrane (IM). Black boxes: transmembrane domains; shaded boxes: HisKA-ATPase domains; ovals: receiver domains; light grey boxes: histidine phosphotransfer (Hpt) domains; white hexagons: output domains, either helix–turn–helix (HTH) or phosphodiesterase (EAL).

In order to test this hypothesis, we systematically investigated protein–protein interactions between relevant domains using bacterial two-hybrid analysis as previously described (Sivaneson *et al*., 43). We tested a total of 14 interactions (Table S1), most of which turned out to be negative. No interaction was observed with PvrS, PvrR or any of the three known single domain Hpt proteins encoded on the *P. aeruginosa* genome (Mikkelsen *et al*., 32). Only the Hpt domain of RcsC displayed a weak, but significant interaction with the receiver domain of RcsB (Fig. 1). We further tested whether a construct containing both the receiver (D) and Hpt domains of RcsC (RcsC-DH) could strengthen the interaction with RcsB, but this was not the case (Fig. 1).

### PvrS increases while RcsC reduces *cupD* gene expression

To further elucidate the role of the sensors, we investigated their influence on *cupD* gene expression. Unlike with the orthologous sensors RocS1 and RocS2, which induce *cupB* and *cupC* gene expression (Sivaneson *et al*., 43), overproduction of RcsC or PvrS did not activate *cupD* transcription (data not shown), which appeared to be strictly dependent on the RcsB response regulator. A reference strain was therefore constructed in which higher levels of RcsB could be obtained. Therefore, the arabinose inducible P_BAD_ promoter was inserted in front of the *rcsB* gene on the chromosome of a strain carrying a *cupD–lacZ* transcriptional fusion in the *att* site (P_BAD_-*rcsB*::DZ, Table 1 and Table S2). In this strain, the level of *rcsB* and therefore *cupD* transcription could be controlled by adding arabinose to the growth medium (data not shown).

**Table 1:** Strains and plasmids used in this study

Strain/plasmid	Relevant characteristics	Resistance^a^	Source
***Escherichia coli***			
DHM1	*cya-854 recA1 gyrA96* (NaI) *thi1 hsdR17 spoT1 rfbD1 glnV44(AS)*		Karimova *et al*. (20)
***Pseudomonas aeruginosa***			
PA14	Wild type	–	Liberati *et al*. (27)
PA14Δ*cupD*	PA14 with a deletion in *cupD1–5*	–	Mikkelsen *et al*. (31)
PA14-*DZ*	PA14 with the *cupD1–lacZ* transcriptional fusion integrated in the *att* site	–	Mikkelsen *et al*. (31)
P_BAD_-*rcsB-DZ*	PA14-*DZ* with the P_BAD_ promoter inserted in front of *rcsB* on the chromosome	–	This study
P_BAD_-*rcsB-DZ*Δ*rcsC*	P_BAD_-*rcsB-DZ* with a clean deletion in *rcsC*	–	This study
P_BAD_-*rcsB-DZ*Δ*pvrS*	P_BAD_-*rcsB-DZ* with a clean deletion in *pvrS*	–	This study
P_BAD_-*rcsB-DZ*Δ*pvrR*	P_BAD_-*rcsB-DZ* with a clean deletion in *pvrR*	–	This study
PA14-*DZ*	PA14 with the *cupD1–lacZ* transcriptional fusion integrated in the *att* site	Tc	This study
PA14-*D1.1Z*	PA14 with the *cupD1.1–lacZ* transcriptional fusion integrated in the *att* site	Tc	This study
PA14-*D1.2Z*	PA14 with the *cupD1.2–lacZ* transcriptional fusion integrated in the *att* site	Tc	This study
**Plasmids**			
miniCTX–*lacZ*	Vector for unmarked integration of transcriptional fusions into the *P. aeruginosa att* site	Tc	Hoang *et al*. (16)
miniCTX-*cupD1–lacZ*	*cupD1* promoter cloned into miniCTX–*lacZ*	Tc	Mikkelsen *et al*. (31)
miniCTX-*cupD1.1–lacZ*	*cupD1* promoter with T^−78^C^−77^C^−76^ mutation cloned into miniCTX–*lacZ*	Tc	This study
miniCTX-*cupD1.2–lacZ*	*cupD1* promoter with T^−158^C^−157^C^−156^ mutation cloned into miniCTX–*lacZ*	Tc	This study
pBBR1-MCS-5	Broad-host-range vector	Gm	Kovach *et al*. (22)
pBBR1-MCS-5-RcsB	*rcsB* cloned into pBBR1-MCS-5	Gm	Mikkelsen *et al*. (31)
pBBR1-MCS-4	Broad-host-range vector	Ap	Kovach *et al*. (22)
pBBR1-MCS-4-RcsC	*rcsC* cloned into pBBR1-MCS-4 (SacI/XbaI)	Ap	This study
pBBR1-MCS-5-RcsC^T506A^	*rcsC* with T506A mutation cloned into pBBR1-MCS-4 (SacI/XbaI)	Ap	This study
pBBR1-MCS-5-RcsC^H502A/T506A^	*rcsC* with H502A and T506A mutations cloned into pBBR1-MCS-4 (SacI/XbaI)	Ap	This study
pBBR1-MCS-5-RcsC^T506A/H1029A^	*rcsC* with T506A and H1029A mutations cloned into pBBR1-MCS-4 (SacI/XbaI)	Ap	This study
pBBR1-MCS-4-PvrS	*pvrS* cloned into pBBR1-MCS-4 (HindIII/XbaI)	Ap	This study
pBBR1-MCS-4-PvrS^H463A^	*pvrS* with H463A mutation cloned into pBBR1-MCS-4 (HindIII/XbaI)	Ap	This study
pBBR1-MCS-4-PvrS^D862A^	*pvrS* with D862A mutation cloned into pBBR1-MCS-4 (HindIII/XbaI)	Ap	This study
pUT18c	Expression vector encoding the T18 fragment of *cyaA*	Ap	Karimova *et al*. (20
pUT18c-RcsB-Rec	pUT18c carrying the Rec domain of RcsB	Ap	This study
pUT18c-PvrR-Rec	pUT18c carrying the Rec domain of PvrR	Ap	This study
pUT18c-TorS	pUT18c carrying the Hpt domain of TorS	Ap	Kulasekara *et al*. (26)
pUT18c-RocA1-Rec	pUT18c carrying the Rec domain of RocA1	Ap	Sivaneson *et al*. (43)
pKT25	Expression vector encoding the T25 fragment of *cyaA*	Km	Karimova *et al*. (20)
pKT25-RcsC-Hpt	pKT25 carrying the Hpt domain of RcsC	Km	This study
pKT25-RcsC-Rec-Hpt	pKT25 carrying the Rec and Hpt domains of RcsC	Km	This study
pKT25-TorR	pKT25 carrying the Rec domain of TorR	Km	Kulasekara *et al*. (26)
pKT25-RocS1-Hpt	pKT25 carrying the Hpt domain of RocS1	Km	Sivaneson *et al*. (43)

a Ap, ampicillin; Km, kanamycin; Sm, streptomycin; Tc, tetracycline; Gm, gentamicin.

**Figure 2:**
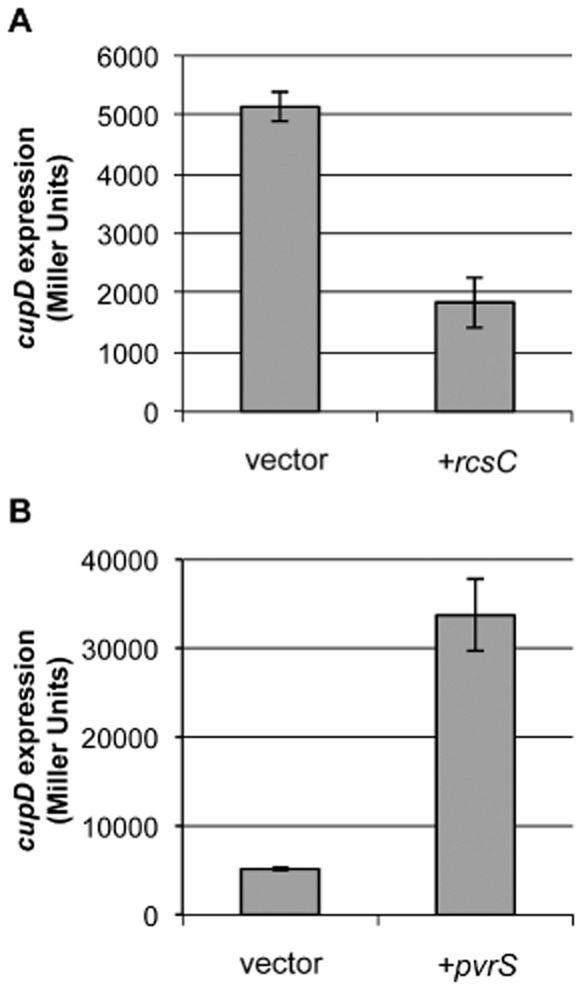
The effect of *rcsC* and *pvrS* overexpression on *cupD* gene expression. β-Galactosidase assays of P_BAD_-*rcsB*::*DZ* carrying empty vector or overexpressing either *rcsC* (A) or *pvrS* (B) as indicated.

Using this reference strain, we investigated the effect of *rcsC* overexpression and found that it reduced *cupD* gene expression by about 2.5-fold (Fig. 2A). This is in agreement with previous results by Nicastro and collaborators, who observed an increase in *cupD* mRNA in an *rcsC* mutant (Nicastro *et al*., 36). Conversely, overexpression of *pvrS* increased the activity of the *cupD* promoter by nearly sevenfold (Fig. 2B), suggesting that the proposed hypothesis had to be revised. To investigate whether either RcsC or PvrS acted via the PvrR response regulator, the respective genes were overexpressed in the reference strain and in an isogenic *pvrR* deletion mutant. This revealed that the effects of the sensors did not depend on *pvrR* (Fig. S1A and B), suggesting that they both act via the RcsB response regulator. We therefore tested whether RcsB activity depends on phosphorylation by replacing the conserved aspartate residue of the receiver domain with the structurally similar asparagine. The resulting protein, RcsB^D71N^, displayed around 100-fold reduced activity compared with the native protein (Fig. S2A). To see whether this could be due to reduced stability of the mutant protein, we engineered pET28a-derivatives encoding His-tagged versions of both RcsB and RcsB^D71N^. Western blot analysis using anti-His antibodies shows that both proteins are produced and stable (Fig. S3). We thus concluded that the conserved Asp71 is required for RcsB activity and this is likely due to the inability of this residue to be phosphorylated. Conversely, PvrR did not appear to require phosphorylation for its activity, since overexpressing a protein lacking the conserved aspartate (PvrR^D57N^) reduced *cupD* transcription by the same amount as the native PvrR protein (around twofold) (Fig. S2B).

PvrS is a hybrid sensor that may require an external histidine phosphotransfer protein (Hpt) in order to transfer a phosphoryl group onto its cognate response regulator. In order to test whether such a protein was required, *pvrS* was overexpressed in the reference strain and in isogenic deletion mutants in either one of the three genes known to encode single domain Hpt proteins, *hptA, hptB* and *hptC*. However, none of these genes were required for PvrS activity (Fig. S1B), suggesting an alternative route of phosphotransfer.

In conclusion, these results are consistent with RcsB activity being controlled by its phosphorylation status, which appears to be antagonistically modulated by two sensors, RcsC and PvrS. This regulation is independent of the RcsB antagonist regulator, PvrR, or of any of the three known single domain Hpt proteins.

### PvrS acts upstream of RcsC

**Figure 3:**
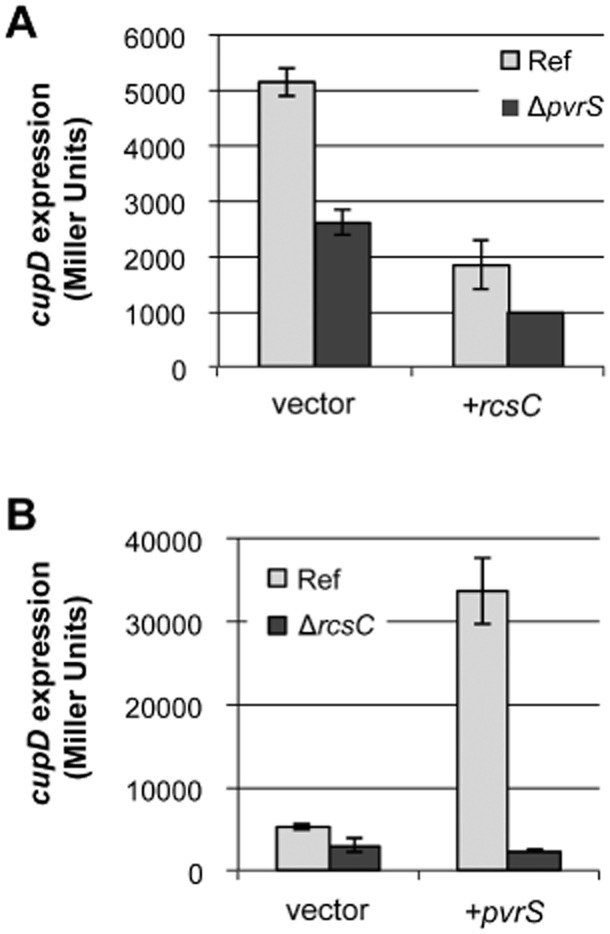
Epistatic analysis of RcsC and PvrS. β-Galactosidase assays of the P_BAD_-*rcsB*::*DZ* reference strain and isogenic deletion mutants carrying empty vector or overexpressing either *rcsC* (A) or *pvrS* (B) as indicated.

Two sensor kinases acting on the same response regulator is reminiscent of the situation with RetS, which negatively regulates phosphorylation levels of the GacA response regulator by inhibiting the activity of the cognate sensor kinase for GacA, namely GacS (Goodman *et al*., 13). To investigate if RcsC might act in a similar manner, and thus directly interfere with PvrS activity, *rcsC* was overexpressed in the reference strain, as well as in an isogenic *pvrS* deletion mutant (Fig. 3A). The *pvrS* mutant carrying the empty vector already displayed a lower *cupD* promoter activity than the corresponding reference strain (about twofold), which likely reflects the loss of the PvrS-dependent positive regulation. However, in both strain backgrounds *rcsC* overexpression led to a 2.7-fold reduction in *cupD* transcription (Fig. 3A), suggesting that RcsC acts independently of PvrS.

In order to test the opposite possibility that PvrS acts via RcsC, *pvrS* was overexpressed in the reference strain and in an isogenic *rcsC* deletion mutant. In this case, the ninefold increase in *cupD* promoter activity upon *pvrS* overexpression in the reference strain was not replicated in the mutant, showing that PvrS acts upstream and likely via RcsC (Fig. 3B).

### PvrS is a kinase, while RcsC is a phosphatase

**Figure 4:**
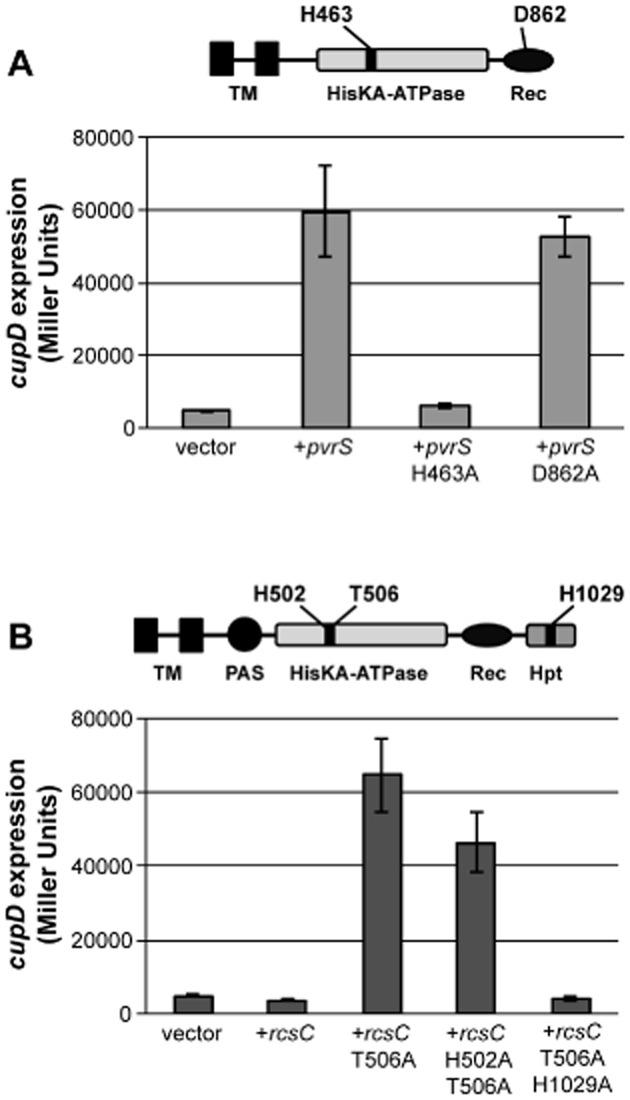
Functional analysis of PvrS and RcsC. β-Galactosidase activity of P_BAD_-*rcsB*::*DZ*Δ*pvrS* (A) or P_BAD_-*rcsB*::*DZ*Δ*rcsC* (B) complemented with constructs encoding either wild-type or mutant proteins of PvrS or RcsC respectively. The domain organization of PvrS and RcsC are shown in (A) and (B) respectively. TM is for transmembrane domain, HisKA-ATPase is the sensor transmitter domain carrying the kinase and ATPase activity, Rec is the receiver domain, Hpt is the histidine phosphotransfer domain, finally PAS is a domain found in several signalling proteins and belongs to the Pfam family PF00989.

Taken together, these data suggest that PvrS is the kinase initiating the phosphorylation cascade in this system, while RscC could act as a phosphatase. This possibility was investigated by site-directed mutagenesis of conserved residues. The kinase activity of PvrS would be predicted to reside in the conserved histidine H463 (Fig. 4A). This residue was substituted for an alanine generating PvrS^H463A^, which was introduced into a *pvrS* deletion mutant for complementation experiments. In contrast to the native PvrS, PvrS^H463A^ overproduction did not lead to any change in *cupD* transcription, confirming that PvrS is likely the kinase (Fig. 4A). Furthermore, to investigate whether the PvrS receiver domain was involved in phosphotransfer, the conserved aspartate was replaced with an alanine, yielding PvrS^D862A^. However, this protein displayed the same activity as the native protein (Fig. 4A), suggesting that phosphotransfer occurs via an alternative route. In all cases we engineered pET28a-derivatives encoding His-tagged versions of PvrS and mutated forms and showed by Western blot analysis that the proteins were produced and stable (Fig. S3).

Previous work has suggested that the putative phosphatase activity of EnvZ-like sensor kinases depends on a residue located only four amino acids from the conserved histidine (Huynh *et al*., 17). To test if the repressing effect of RcsC could be due to phosphatase activity, the threonine residue in this position was replaced by an alanine. Overproduction of the resulting RcsC^T506A^ resulted in a substantial activation of transcription compared with the vector control (Fig. 4B), which is consistent with the T506A mutation resulting in the loss of phosphatase activity. The increase in transcriptional activity was not due to a previously masked kinase activity, since an additional mutation in the conserved histidine (RcsC^H502A–T506A^) made little difference to RcsC activity. Instead, since PvrS activity was shown to depend on RcsC (Fig. 3B), this could suggest that RcsC had a second activity as a phosphorelay. Indeed, introduction of a mutation in the conserved histidine of the Hpt phosphotransfer domain (RcsC^T506A–H1029A^) abolished the activity of the protein (Fig. 4B).

### The *cupD1* promoter has a putative RcsB binding site

**Figure 5:**
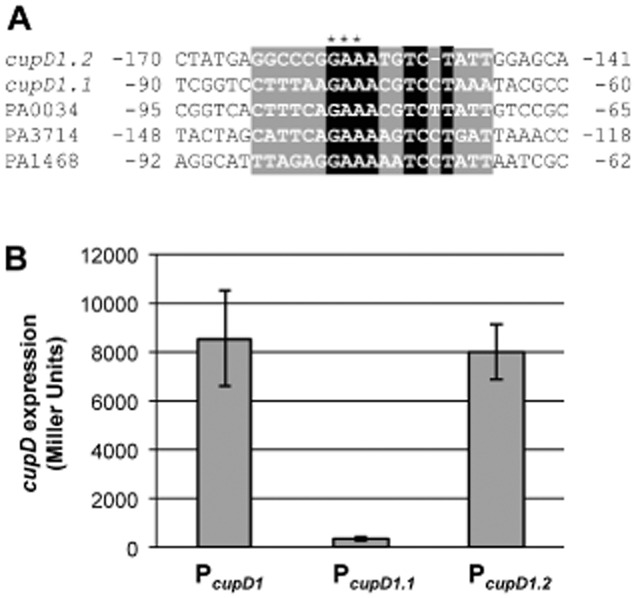
Identification of putative RcsB binding sites.A. Alignment of target promoter sequences. Numbers indicate distance from +1. Black or grey shading indicates putative conserved or partly conserved nucleotides respectively. Asterisks indicate nucleotides in the *cupD1* promoter that are mutated in (B) (GAA→TTC).B. β-Galactosidase assays of the P_BAD_-*rcsB* reference strain carrying either wild-type or mutated *cupD–lacZ* promoter fusions in the *att* site.

An analysis of the *cupD1* promoter identified two putative RcsB binding sites at around −155 and −75 relative to the open reading frame (Fig. 5A). The latter of these was suggested in a previous publication by Nicastro *et al*. (36) based on similarities to the binding sites of homologous response regulators in other bacteria. To test which of the two RcsB binding sites was correct, *cupD1–lacZ* transcriptional fusions (*cupD1.1* and *cupD1.2*) with mutations in nucleotides shown to be crucial for binding in the similar RcsAB box of *Escherichia coli* (Wehland and Bernhard, 53) were integrated onto the chromosome (Fig. 5A). This showed that the three nucleotides G^−78^A^−77^A^−76^ were crucial for *cupD1* promoter function (Fig. 5B), whereas mutation of G^−158^A^−157^A^−156^ had no effect.

### *rcsB* overexpression inhibits biofilm formation during growth in microfermentors

**Figure 6:**
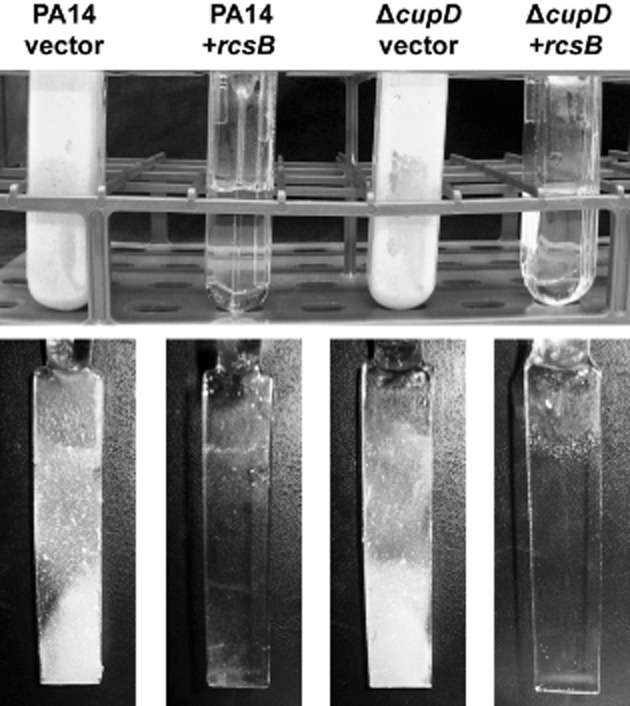
Biofilms formation in microfermentors. PA14 or PA14Δ*cupD* carrying empty vector or overexpressing *rcsB* as indicated. Top: microfermentors after 4 days growth; bottom: biofilms formed on the glass spatulas.

We have previously shown that overexpression of *rcsB* leads to a number of phenotypes including increased attachment (Mikkelsen *et al*., 31). Intriguingly, these phenotypes were only partly dependent on the production of CupD fimbriae, and a loss of attachment was observed when *rcsB* was overexpressed in a *cupD* deletion mutant, suggesting that under certain circumstances RcsB could promote dispersal at the expense of attachment. We further assessed the impact of *rcsB* overexpression when bacteria were grown in dynamic conditions and not in static conditions as done in our previous work (Mikkelsen *et al*., 31). For this purpose, we used an *in vitro* model for biofilm formation in microfermentors as previously described (Valle *et al*., 47). When cells were grown in these conditions (see *Experimental procedures*) overexpression of *rcsB* from pBBR1MCS-5-*rcsB* prevented biofilm formation of the parental strain, as well as of the *cupD* mutant (Fig. 6). This observation thus suggests either that the phosphorylation level and activity of RcsB varies depending on the growth conditions, or that RcsB controls other genes that are involved in balancing biofilm formation and dispersal.

### RcsB induces biofilm dispersal in a PvrR-dependent manner

**Figure 7:**
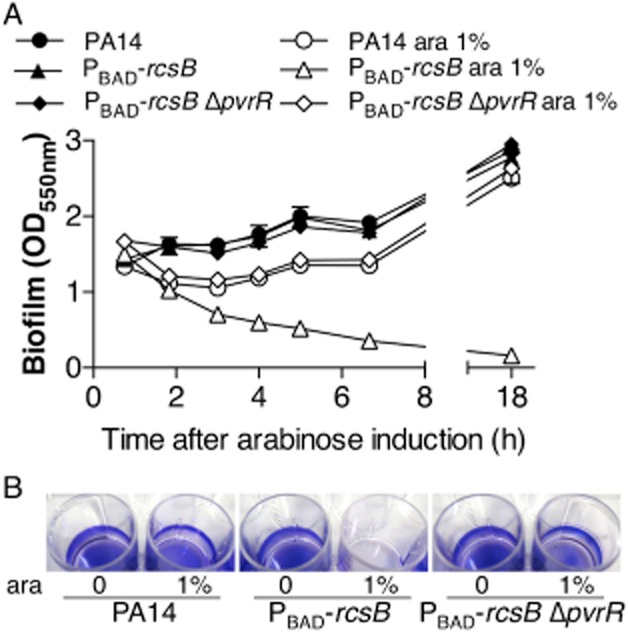
RcsB-dependent dispersal of pre-formed biofilms requires *pvrR*. Biofilms were grown in 24-well plates in static conditions for 6 h, and arabinose was added to induce *rcsB*. Biofilm dispersal was then monitored using crystal violet (CV) staining.A. Time-course of dispersal after addition of arabinose at 45 min, 2 h, 3 h, 4 h, 5 h, 7 h and 18 h. Error bars represent standard deviation (*n* = 2).B. Images of CV stained biofilms in wells after 18 h *rcsB* induction with arabinose.This figure is available in colour online at wileyonlinelibrary.com.

Because of its dual role in biofilm formation, the RcsB regulon was investigated in a microarray analysis that compared PA14 either overexpressing *rcsB* or carrying an empty vector control. An obvious target which was readily identified was the *pvrR* gene which was upregulated nearly fourfold upon *rcsB* overexpression (Table S3). An increase in PvrR levels should indeed promote dispersal since we previously showed that overexpression of *pvrR* from a plasmid prevented biofilm formation under static conditions (Mikkelsen *et al*., 31). However, with a plasmid-based system it was not possible to fully repress the expression of *rcsB* and thereby *pvrR* in a developing biofilm. To systematically assess the contribution of PvrR to biofilm dispersal we therefore introduced the P_BAD_ promoter in front of *rcsB* and then engineered an isogenic *pvrR* mutant and tested these strains for dispersal in a static biofilm system (see *Experimental procedures*). Biofilms were grown for 6 h, and arabinose was then added to induce *rcsB* expression. Whereas arabinose addition did not impact the biofilm formed by the PA14 wild type, the P_BAD_-*rcsB* biofilm was fully dispersed within 18 h (Fig. 7). However, this dispersal effect was completely abolished in the corresponding *pvrR* mutant, suggesting that PvrR is strictly required for the RcsB-dependent dispersal process. This effect was observed using standard crystal violet (CV) staining in 24-well plates, and the dispersal kinetic was monitored from 45 min post-arabinose induction up to 18 h (Fig. 7). We also investigated the structures of LIVE/DEAD stained biofilms grown on glass-bottom 24-well plates (Fig. S4). Without arabinose, biofilms almost fully covered the surface and displayed aggregate structures resembling young microcolonies comprised of mainly live cells (Fig. S4). In contrast, arabinose-dependent *rcsB* induction in the P_BAD_-*rcsB* biofilm showed only a few attached live cells, sparsely distributed across the surface and there were no cell aggregates, thus indicative of dispersal. Addition of arabinose to the *pvrR* mutant led to a biofilm phenotype that was identical to the wild type with arabinose (Fig. S4).

### RcsB controls other regulators on the core genome

We further analysed the microarray data to get an understanding of the complete RcsB regulon. In addition to the *cupD* and *rcs/pvr* gene clusters, a few genes on the PAPI-1 pathogenicity island were found to be significantly modulated (Table S3). For example the *pilL2* and *pilP2* genes, which are involved in the assembly of the type IVb pili required for transferring the PAPI-1 island between strains, were two- to threefold downregulated (Carter *et al*., 3; Filloux, 8). More importantly, we observed that several genes on the core genome were significantly modulated upon RcsB overproduction (Table S3). Genes encoding inner membrane proteins or proteins with an export signal appeared to be over-represented, especially among the downregulated genes. Furthermore, a number of genes related to other appendages were downregulated, such as the *cupE* fimbrial genes (about 2.5-fold) (Giraud *et al*., 10), the *cupB5* adhesin gene (about fourfold) (Ruer *et al*., 41), and the RocS1 sensor that activates the *cupB* gene cluster (about twofold). This could be related to the stress of over-producing CupD fimbriae. Furthermore, a large group of downregulated genes (two- to threefold) was related to the type III secretion system (Table S3). These include effectors-encoding genes (*exoT* and *exoY*), genes encoding components of the T3SS machinery, *pscS*, *pscF* and *pscK*, the translocator-encoding gene *popB* and the gene encoding the needle tip protein, *pcrV*. A downregulation of the T3SS in response to an activation of the *cupD* fimbrial cluster is in agreement with the antagonistic regulation of genes involved in biofilm formation and virulence (Goodman *et al*., 12; Moscoso *et al*., 34).

Interestingly, a number of genes were strongly induced by RcsB overproduction, and Table 2 lists those that displayed a more than fourfold induction. Although many of these genes encode proteins with no known function, we identified putative RcsB binding sites in at least three promoter regions in addition to *cupD* (Fig. 5A). PA0034 and PA3714 encode orphan response regulators with an N-terminal receiver domain and a C-terminal helix–turn–helix output domain. Furthermore, PA0034 is in an operon with *hptC* (PA0033), which encodes one of the three known single domain Hpt proteins of *P. aeruginosa* (Rodrigue *et al*., 40). PA1468 encodes a hypothetical protein with no predicted conserved domain. However, structural modelling using Phyre (Kelley and Sternberg, 21) showed a 100% match to the receiver domain of the *E. coli* RcsC protein (Fig. S5). The three genes with predicted RcsB binding sites therefore all encode putative response regulators. Also PA0267 encodes an orphan response regulator with an N-terminal receiver domain, but no RcsB binding site was identified in this case.

**Table 2:** Microarray analysis

PA No.	Fold change	Gene	Gene product
PA14_59770	112.8	*rcsB*	Two-component response regulator
PA14_59710-60^*^	6.7–59.5	*cupD1–5*	CupD fimbrial cluster
PA0027	5.2		Hypothetical protein
PA0033	10.4	*hptC*	Histidine phosphotransfer protein HptC
PA0034^*^	8.6		Probable two-component response regulator
PA0267	6.8		Hypothetical protein
PA0746	5.4		Probable acyl-CoA dehydrogenase
PA1468^*^	9.9		Hypothetical protein
PA1571	4.5		Hypothetical protein
PA1664	9.4	*orfX*	OrfX
PA2075	4.1		Hypothetical protein
PA2111	6.5		Hypothetical protein
PA2553	6.0		Probable acyl-CoA thiolase
PA2554	5.9		Probable short-chain dehydrogenase
PA2557	4.8		Probable AMP-binding enzyme
PA2605	7.6		Conserved hypothetical protein
PA3179	4.3		Conserved hypothetical protein
PA3714^*^	4.5		Probable two-component response regulator
PA4208	8.6	*opmD*	Probable outer membrane protein precursor

Genes that were significantly induced (fold change ≥4, *P* ≤ 0.05) in PA14 overexpressing *rcsB* compared with an empty vector control.

A full list of significantly modulated genes can be seen in Table S3.

For clarity, PAO1 locus numbers have been used for genes outside the pathogenicity island.

Asterisks indicate genes with putative RcsB binding sites in the promoter.

## Discussion

The RcsB–RcsC TCS was annotated by homology to the equivalent proteins in *Salmonella* and *E. coli* (He *et al*., 15; Majdalani and Gottesman, 28; Clarke, 5). No previous studies have addressed the function of the PvrS sensor, but the PvrR response regulator was first identified as a phenotype variant regulator that upon overexpression could reverse hyperbiofilm antibiotic-resistant variants back to the wild-type phenotype (Drenkard and Ausubel, 7). However, this effect is unlikely to be specific, since PvrR is an active phosphodiesterase that hydrolyses c-di-GMP (Kulasakara *et al*., 24; Meissner *et al*., 30; Chung *et al*., 4) and therefore is likely to elicit a global response upon overexpression.

**Figure 8:**
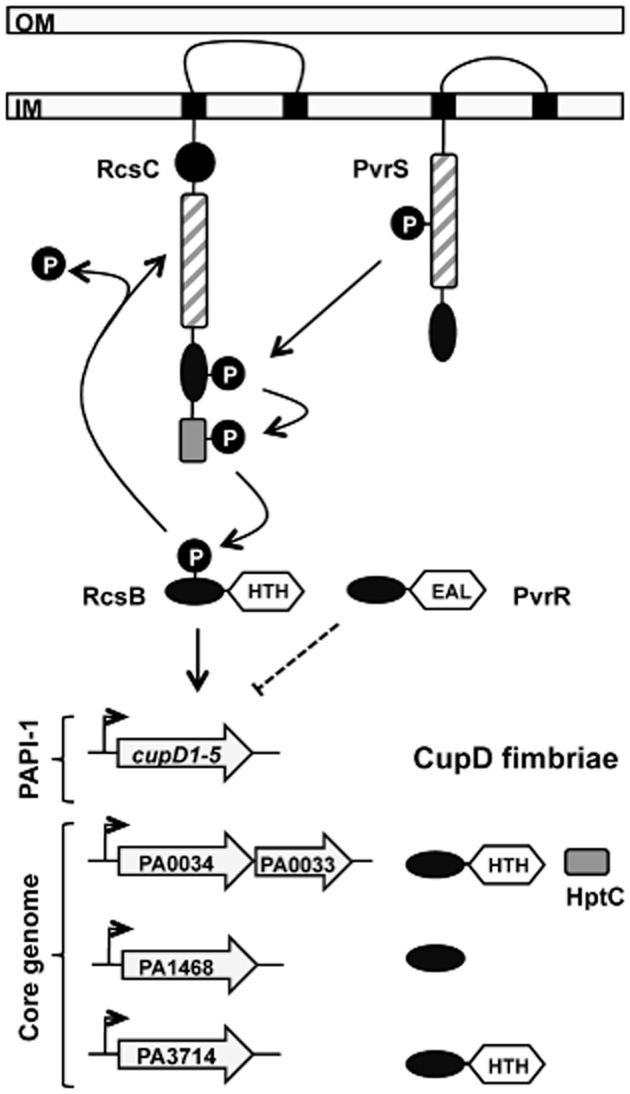
Regulation model. The *cupD* gene cluster is inversely regulated by the response regulators RcsB and PvrR. RcsB is activated by the PvrS kinase, while RcsC acts as a phosphorelay and a phosphatase. In addition to its primary target, the *cupD* promoter, RcsB also activates the expression of other regulatory proteins encoded on the core genome. RcsB also positively influences expression of *pvrR*, which downregulates production of CupD fimbriae and reduces biofilm formation. Shaded boxes: HisKA-ATPase domains; ovals: receiver domains; light grey boxes: histidine phosphotransfer (Hpt) domains; white hexagons: output domains, either helix–turn–helix (HTH) or phosphodiesterase (EAL).

We have previously shown that the RcsB and PvrR response regulators inversely control expression of the *cupD* gene cluster (Mikkelsen *et al*., 31). This is similar to the RocA1 and RocR response regulators that antagonistically control the expression of the *cupC* gene cluster (Kulasekara *et al*., 26; Ruer *et al*., 42; Sivaneson *et al*., 43). However, unlike previously suggested (He *et al*., 15; Mikkelsen *et al*., 31), the Rcs/Pvr proteins do not form two sensor-response regulator pairs. Instead, we found that PvrS is a positive regulator of *cupD* transcription, while RcsC is a negative regulator (Fig. 8). We further showed that PvrS is likely a kinase that activates the RcsB response regulator, since PvrS activity was abolished by mutation of the conserved histidine (H463A). While it cannot be excluded that the receiver domain has some activity, mutation of the aspartate (D862A) did not influence PvrS function in the conditions tested (Fig. 4A). Furthermore, epistatic analysis indicates that the positive regulation by PvrS requires the presence of RcsC, which therefore likely acts as a phosphorelay in addition to its role as a negative regulator (Fig. 8). The role of a sensor-like component as phosphorelay has already been documented in several instances. This is the case of the receiver domain of the *E. coli* RcsD (also known as YojN), which however lacks the conserved histidine residue in its transmitter domain (Majdalani and Gottesman, 28). The CblRST regulatory system in *Burkholderia cepacia* controls the expression of genes encoding components required for the formation of so-called cable pili (Tomich and Mohr, 45,b). In this case both sensors, CblS and CblT display a kinase activity. Interestingly, the Hpt domain of the ClbT sensor relays phosphate to the ClbR response regulator either from ClbT or from ClbS.

While most TCS research focuses on phosphorylation, phosphotransfer and positive regulation, negative regulation is essential, both for resetting the system and for avoiding cross-talk (Huynh *et al*., 17; Whitworth, 54). This is partly achieved by autodephosphorylation of the response regulator receiver domain, which probably occurs at a fixed rate for a given protein (Bourret, 1). However, the majority of transmitter domains are bifunctional and also contribute to dephosphorylation in a signal-dependent manner. This regulated dephosphorylation sets the balance between positive and negative control and therefore determines the output (Huynh and Stewart, 18). Previous research has suggested that the phosphatase function of HisKA family sensors resides in an E/DxxT/N motif immediately adjacent to the conserved histidine of the transmitter domain (Huynh *et al*., 17). Mutation of the corresponding T506 of RcsC led to a dramatic increase in *cupD* transcription. This does not seem to be due to a masked kinase activity, since an additional H502A mutation did not alter the activity of the *cupD* reporter (Fig. 4B). The RcsC sensor is likely not a kinase, but instead displays a strong phosphatase activity. It is well known that sensor kinases can possess multiple activities and carry out autophosphorylation, phosphotransfer, as well as dephosphorylation (Whitworth, 54). However, it is highly interesting that in the PvrS–RcsC–Rcs phosphorylation cascade, these functions appear to be split between the two sensor proteins with PvrS being the kinase and RcsC the phosphatase.

Additional negative regulation of the *cupD* genes is provided by the PvrR response regulator (Mikkelsen *et al*., 31). The bacterial two-hybrid experiments could not identify any interaction between PvrR and either one of the two sensors, RcsC or PvrS. This suggested that either PvrR is phosphorylated by an unknown sensor or PvrR phosphorylation is not required for its activity. To investigate this, a mutation was introduced into the conserved aspartate of the receiver domain. This mutation did not alter the activity of the protein, suggesting that PvrR does not require phosphorylation for its function (Fig. S2B). Not all response regulators depend on phosphorylation (Bourret, 1). Examples of this are RcsA in *E. coli*, which has a highly degenerate receiver domain (Majdalani and Gottesman, 28), and VpsT in *Vibrio cholerae*, the receiver domain of which oligomerizes upon c-di-GMP binding (Krasteva *et al*., 23). However, the PvrR receiver domain does not appear to be degenerate, nor does it possess the extension that drives the c-di-GMP mediated dimerization of VpsT. Expression of *pvrR*, as well as *pvrS, rcsC* and *rcsB*, is induced by RcsB overproduction (Table S3 and data not shown). This observation indicates that RcsB positively controls two antagonistic functions, which on the one hand increases production of CupD fimbriae and therefore biofilm formation, and on the other hand increases levels of PvrR resulting in lower amount of c-di-GMP and therefore biofilm dispersal. The PvrR-dependent induction of biofilm dispersal is demonstrated here by stimulating the detachment of pre-formed biofilm by induction of the *rcsB* gene (Fig. 7). This antagonism between increase in fimbrial structures and decrease in c-di-GMP levels is not unprecedented since it is a situation similar to what has been observed with the Roc system in which the RocR phosphodiesterase counteracts the production of CupB and CupC fimbriae (Kulasekara *et al*., 26; Ruer *et al*., 42). However, whether PvrR activity is solely regulated by transcription, or whether other control mechanisms are in place, is currently unknown.

We have also looked for alternative RcsB targets using microarrays. In addition to the *cupD* gene cluster on the PAPI-1 pathogenicity island, a substantial number of genes on the core genome were significantly modulated. Several of the most highly induced genes encode hypothetical proteins (Table 2). However, three genes were particularly interesting, since they appeared to have RcsB binding sites in their respective promoter sequences: PA3714 and PA0034 encode putative DNA-binding orphan response regulators, and PA0034 is in an operon with the *hptC* phosphorelay gene (PA0033). PA1468 encodes a hypothetical protein and is in an operon with a multicopper oxidase. The primary sequence gives little clue about its function, but structural modelling revealed that it is most likely a CheY-like receiver domain (Fig. S5) with five α-helices surrounding a five-stranded parallel β-sheet (Cho *et al*., 6). Such single domain response regulators can play important roles in chemotaxis, as allosteric regulators of sensor kinases, or as spatial regulators, as has recently been shown in *Caulobacter crescentus* (Jenal and Galperin, 19; Paul *et al*., 37). Preliminary experiments showed that single deletions of any of these regulatory genes, does not influences the RcsB-dependent phenotype associated with biofilm formation or dispersal (data not shown). Future work will aim to investigate the role of these three regulatory genes within the characterized RcsB regulon, particularly with respect to biofilm formation and the cytotoxicity associated with the T3SS.

Our present work places the RcsB response regulator in the centre of a complex regulatory network. Two sensors of opposing function regulate its activity: PvrS that probably acts as a kinase, and RcsC that plays a dual role, first as a possible phosphorelay and second as a phosphatase that resets the system. Unlike most TCSs, this atypical system has separated the kinase and phosphatase activity, possibly in order to integrate different types of input stimuli. The primary function of the regulatory system is to control the production of CupD fimbriae, which contribute to attachment. However, it also controls the expression of PvrR, the phosphodiesterase activity of which can induce the dispersal of pre-formed biofilms. The physiological relevance of this seemingly antagonistic behaviour might be that once the biofilm is formed, a small part of the population can disperse thus allowing a subpopulation of planktonic cells to further colonize the host or the environment. Moreover, other regulators on the core genome, the phosphodonors and downstream targets of which are currently unknown, are also directly controlled by RcsB and therefore the subtlety and complexity of the response needs further investigation. To our knowledge, this is the first regulation network of its kind described in *P. aeruginosa* and related species. Such work is also a clear demonstration that not only molecular devices, but also sophisticated regulatory systems, can be acquired by bacteria by horizontal gene transfer. Future work will aim at confirming the effective phosphotransfer between the components of these TCSs by using state of the art biochemical approaches.

## Experimental procedures

### Bacterial strains, plasmids and growth conditions

Bacterial strains and plasmids are listed in Table 1 and supplementary Table S2. Unless otherwise stated, bacteria were grown in LB broth or on LB agar at 37°C. Antibiotics were used at the following concentration: For *E. coli*, ampicillin, kanamycin, gentamicin and streptomycin 50 μg ml^−1^, tetracycline 15 μg ml^−1^. For *P. aeruginosa* selection and maintenance respectively: carbenicillin 500/300 μg ml^−1^, gentamicin 150/100 μg ml^−1^, tetracycline 200 μg ml^−1^ and streptomycin 2 mg ml^−1^. Transfer of plasmids into *P. aeruginosa* was achieved by triparental mating using the mobilizing plasmid pRK2013.

### Construction of bacterial strains and plasmids

Oligonucleotides for overexpression and mutator constructs are listed in Table S4. PCR products were cloned into the pCR2.1-TA cloning vector and sequenced, prior to subcloning into the relevant broad-host-range or suicide vector. His-tagged constructs were generated by PCR amplification of wild-type or mutant templates, cloning into pCR-Blunt II-TOPO and subcloning into the pET28a expression vector for N-terminal His-tagging. Deletion mutants were generated as previously described (Ruer *et al*., 42; Mikkelsen *et al*., 31) using the pKNG101 suicide vector and selection on streptomycin followed by 5% sucrose. To generate a strain with an inducible *rcsB* gene, the region containing *araC* and the P_BAD_ promoter was amplified from the pJN105 plasmid (Newman and Fuqua, 35) and inserted in between the 5′ end of the *rcsC* gene (∼550 bp) and the 3′ end of the *rcsB* gene (∼530 bp). This mutator fragment was then cloned into the pKNG101 suicide vector and used to generate the reference strain P_BAD_-*rcsB-DZ*. Transcriptional fusions were subcloned into miniCTX–*lacZ*, introduced into *P. aeruginosa* by bi-parental mating using *E. coli* SM10 and integrated into the *att* site as previously described (Hoang *et al*., 16). Site-directed mutagenesis was carried out on constructs in pCR2.1 using the QuickChange II Site-Directed Mutagenesis Kit (Stratagene) following the supplier's instructions.

### Bacterial two-hybrid analysis

Bacterial two-hybrid analysis was carried out as previously described. Briefly, gene fragments encoding protein domains of interest were cloned into plasmids pKT25 and pUT18c (Karimova *et al*., 20). Recombinant plasmids were then co-transformed into *E. coli* DHM1, and independent transformant colonies were inoculated into overnight cultures that were spotted onto MacConkey agar supplemented with antibiotics, IPTG and 1% maltose. Positive interactions were identified as dark red colonies. The strength of the interaction was quantified by resuspending the cells thoroughly in 0.9% saline and measuring β-galactosidase activity as described below.

### β-Galactosidase assays

In agreement with previously published data (Nicastro *et al*., 36), the highest expression levels of the *cupD* gene cluster were achieved during growth on solid medium at 30°C. Colonies of fresh transconjugants (*n* ≥ 3) were therefore patched onto M63 minimal agar supplemented with 1 mM MgSO_4_, appropriate antibiotics and X-gal for visualization and incubated at 30°C overnight. Cells were scraped off the plates and resuspended thoroughly in 0.9% saline for OD_600_ measurements, then sedimented and β-galactosidase measurements were carried out using the Miller method as previously described (Miller, 33).

### Microarray analysis

Plasmids were introduced into the PA14 parental strains by triparental mating. Overnight cultures from three independent transconjugants were inoculated into M63 medium and grown at 37°C with shaking to an OD_600_ of 1, then harvested into RNA*later* (Ambion). RNA was extracted using the RNeasy extraction kit (Qiagen), DNA was removed using the Turbo DNA-free kit (Applied Biosystems), and the RNA was re-purified using the RNeasy kit, following the supplier's protocol. Microarray analysis was carried out as previously described (Rampioni *et al*., 39; Sivaneson *et al*., 43) using custom made arrays from Oxford Gene Technology (Oxford, UK) containing oligos for all PAO1 genes and small RNAs, as well as any additional genes found in PA14. Data were normalized using Lowess, and genes displaying a fold change ≥ 2 and a corrected *P*-value ≤ 0.05 were considered significantly modulated.

### Biofilm assay in microtitre plates

Biofilms of *P. aeruginosa* PA14, P_BAD_-*rcsB-DZ* or P_BAD_-*rcsB-DZ*Δ*pvrR* strains were grown from a 1:200 dilution of an overnight culture in LB into 1 ml M9 minimal medium (containing 48 mM Na_2_HPO_4_, 22 mM KH_2_PO_4_, 9 mM NaCl, 19 mM NH_4_Cl, 2 mM MgSO_4_, 100 μM CaCl_2_, pH 7.2) with 20 mM glutamate as a carbon source, in tissue-culture treated 24-well plates (BD) incubated without shaking at 30°C. After 6 h of growth, 1% arabinose was added or not to wells in duplicate, and the plates were further incubated for up to 18 h. Biofilm biomass was analysed using crystal violet staining. After washing once with phosphate-buffered saline (PBS), 1 ml crystal violet stain (0.2% crystal violet, 1.9% ethanol and 0.08% ammonium oxalate in PBS) was added to the wells and the plates were incubated on the bench for 20 min before washing twice with PBS. After photographing the stained biofilms, the crystal violet was redissolved with 1 ml 100% ethanol and quantified by measuring the OD_550_ of the homogenized suspension. OD measurements of control wells without bacteria at the beginning of the experiment were subtracted from all values.

Biofilms were also grown in glass bottom 24-well plates (MatTek Corporation, Ashland MA, USA) for 6 h and treated with 1% arabinose for 18 h as described above. Then biofilms were rinsed twice with PBS before being stained with LIVE/DEAD *Bac*Light bacterial viability kit reagents (Molecular Probes) and visualized by using an inverted widefield microscope (Zeiss Axio Observer).

### Biofilm assay in microfermentors

Biofilms of *P. aeruginosa* PA14 or PA14Δ*cupD* strains harbouring the plasmids pBBR1-MCS-5 or pBBR1-MCS-5-*rcsB* were grown under turbulent continuous flow-through conditions in glass microfermentors (Valle *et al*., 47). After an initial attachment period of 1 h at 30°C without flow nor aeration, a flow of fresh M9 minimal medium containing 2 mM glucose and supplemented with 100 μg ml^−1^ gentamicin was set at a constant rate of 0.8 ml min^−1^ and aeration at 40 kPa and 0.1 l min^−1^. Biofilm formation on the microfermentor spatula was assessed after 4 days of growth. The experiment was repeated three times with similar results.
