# Supplementary material for: The pathogenicity island encoded PvrSR/RcsCB regulatory network controls biofilm formation and dispersal in Pseudomonas aeruginosa PA14
Source: Mol Microbiol. 2013 Jun 28;89(3):450–63. doi: 10.1111/mmi.12287 (PMC3842833; doi:10.1111/mmi.12287)
Supplement: Supplementary file 1 [file mmi0089-0450-sd1.pdf]

The pathogenicity island encoded PvrSR/RcsCB regulatory network controls biofilm formation and dispersal in *Pseudomonas aeruginosa* PA14

Helga Mikkelsen,[1] Kailyn Hui,[1] Nicolas Barraud,[2] and Alain Filloux[1,*]

[1]Imperial College London, Department of Life Sciences, MRC Centre for Molecular Bacteriology and Infection, South Kensington Campus, Flowers Building, SW7 2AZ London, United Kingdom; [2]School of Biotechnology and Biomolecular Sciences and Centre for Marine Bio-Innovation, University of New South Wales, Sydney, New South Wales 2052, Australia

Supplementary Information

Supplementary Figures

Figure S1: RcsC and PvrS are independent of PvrR, HptA, HptB and HptC

Figure S2: RcsB requires phosphorylation, but PvrR does not

Figure S3: Mutant proteins of RcsB and PvrS are stable

Figure S4: RcsB-dependent biofilm dispersion requires *pvrR*

Figure S5: PA14_45450 is structurally similar to the *E. coli* RcsC receiver domain

Supplementary Tables

Table S1: Protein-protein interactions tested by bacterial two-hybrid

Table S2: Supplementary strains

Table S3: Full list of genes modulated by RcsB overproduction

Table S4: Oligonucleotides used in this study

Supplementary Reference

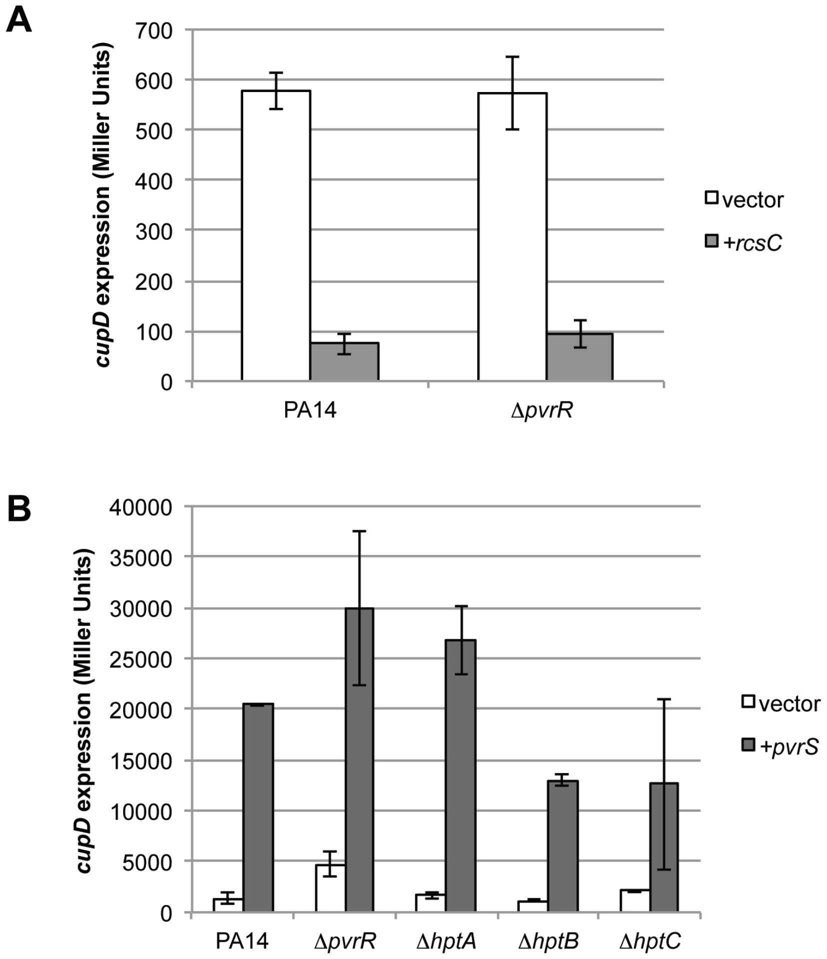

Fig. S1. β-galactosidase assays of P_{BAD}-*rcsB*::*DZ* or isogenic deletion mutants carrying either empty vector or overexpressing *rcsC* (A) or *pvrS* (B) as indicated. Strains were grown on M63 agar supplemented with 0.01% arabinose.

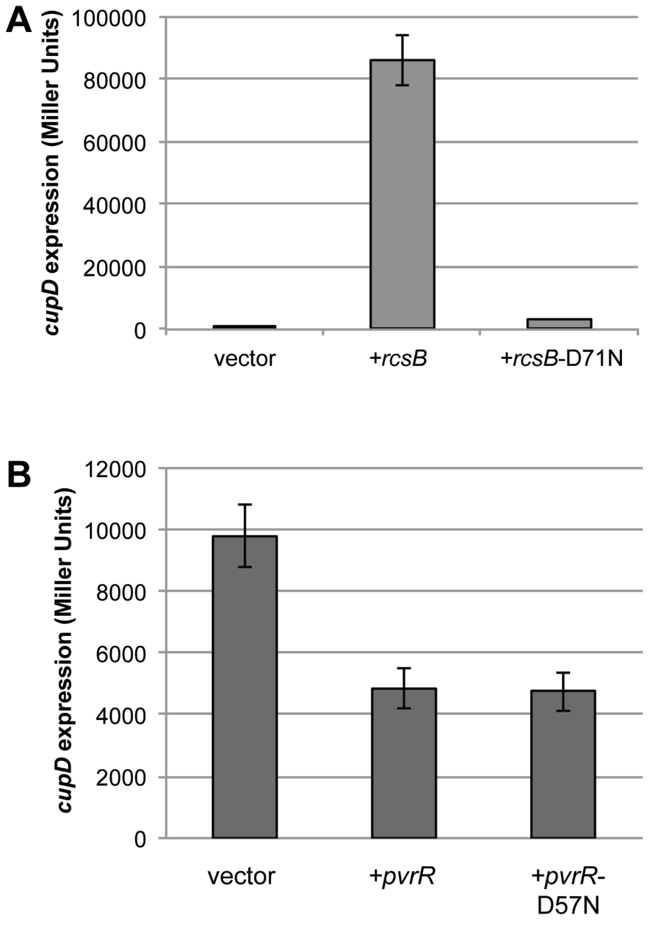

Fig. S2. RcsB requires phosphorylation, while PvrR does not. β-galactosidase assays of PA14::*cupD-lacZ* (**A**) or PA14::*pBAD-rcsB*::*cupD-lacZ* (**B**) carrying empty vector or overexpression constructs as indicated. Cells were grown on M63 minimal agar supplemented with appropriate antibiotics and 0.05% arabinose in (B) to activate the *cupD* gene cluster.

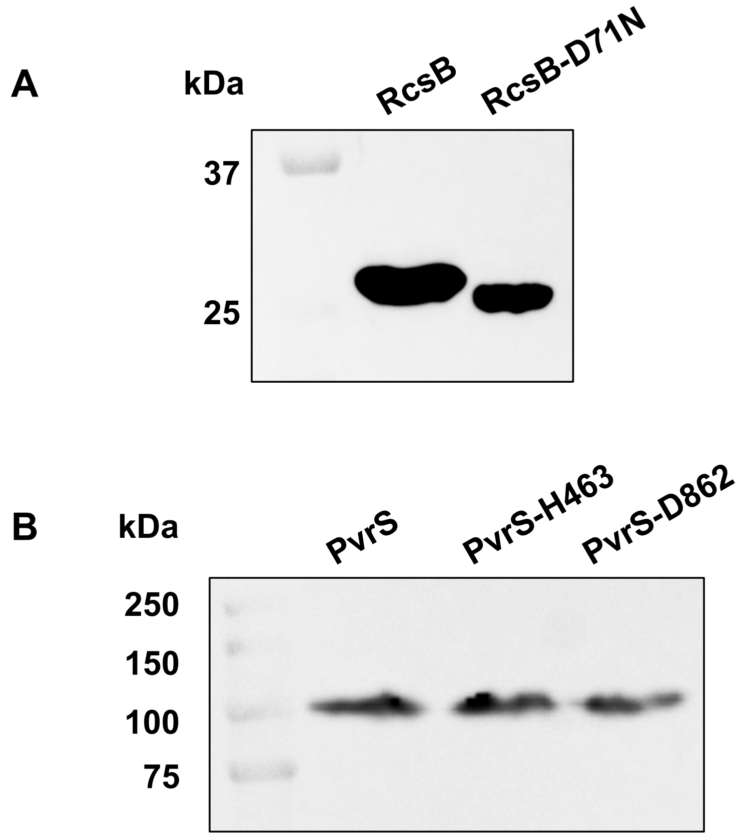

Fig. S3. Mutant proteins of RcsB and PvrS are stable. Immunoblots of wild type and mutant proteins of RcsB (A) and PvrS (B) as indicated. Whole cell extracts were separated on SDS-PAGE gels, and immunoblotting was carried out as previously described (Hachani *et al.*, 2011). The primary antibody was polyclonal anti-His antibody (Sigma), and the secondary was peroxidase-conjugated anti-mouse IgG (Sigma). Both antibodies were used at a dilution of 1:5000.

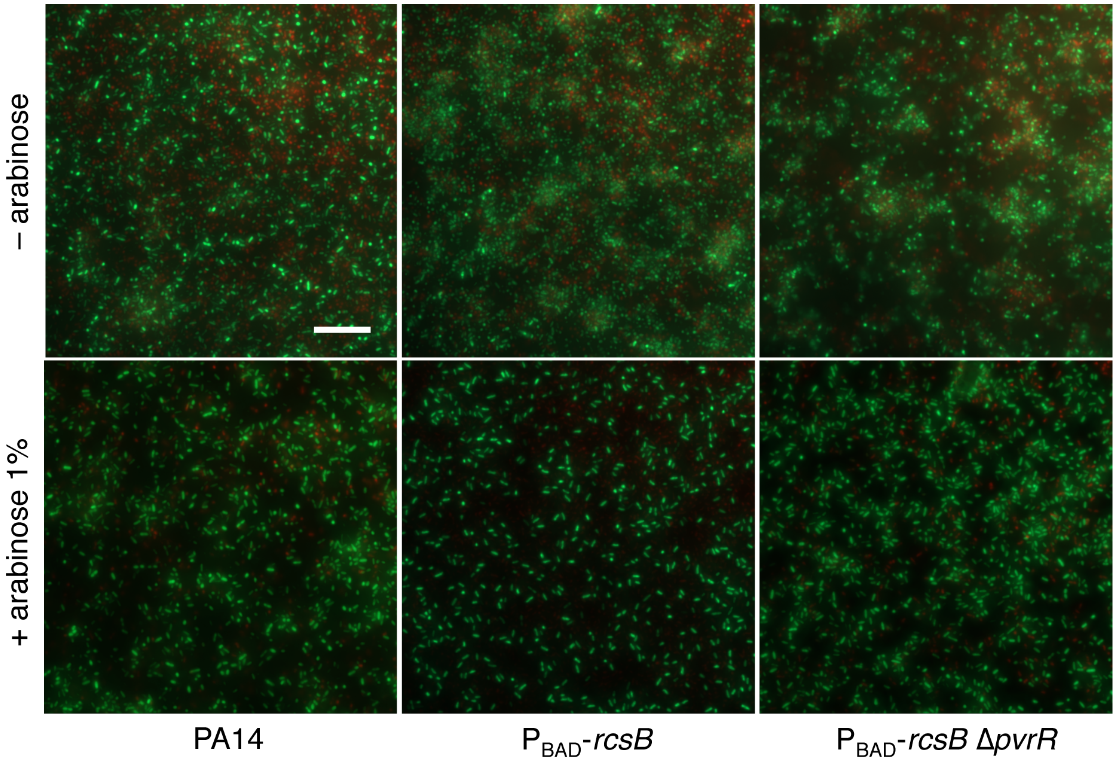

Fig. S4. RcsB-dependent biofilm dispersion requires *pvrR*. Microscopic images of LIVE/DEAD stained biofilms at the bottom of 24-well plates after 18 h *rcsB* induction with arabinose. Live cells are stained green and dead cells are red. Scale bar: 10 μm.

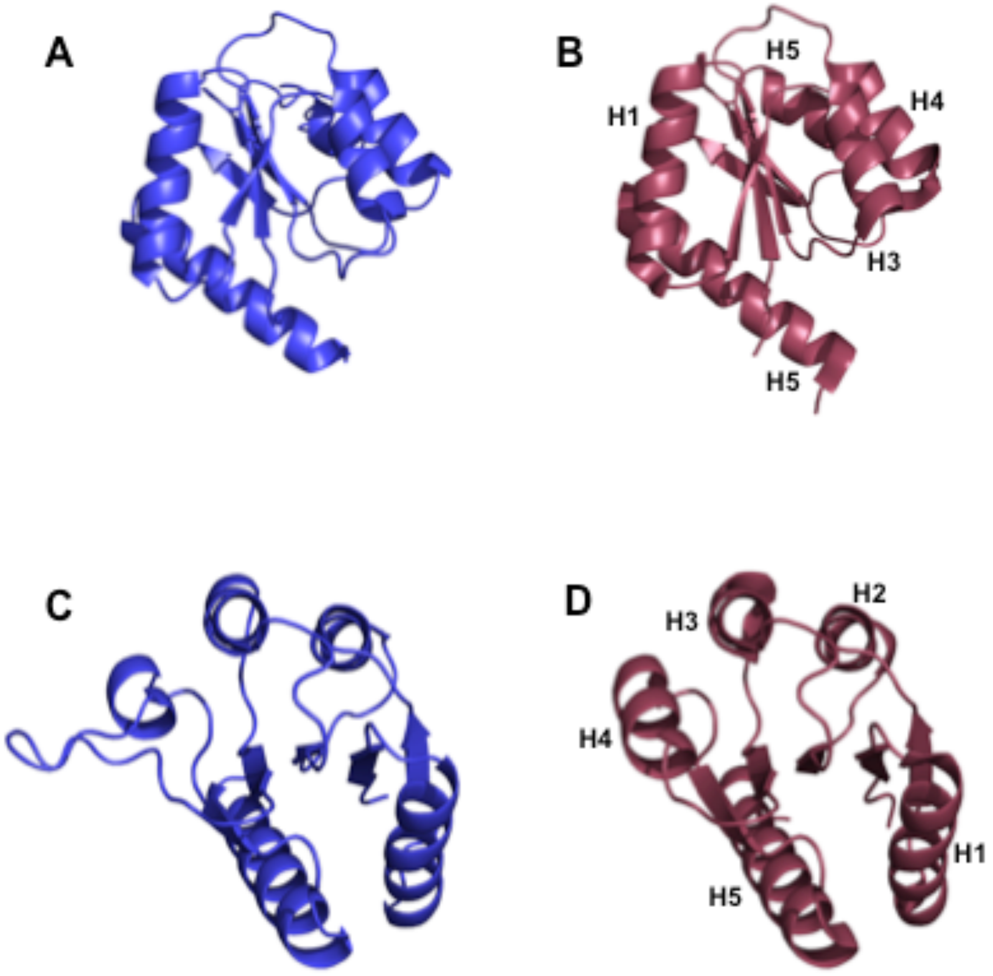

Fig. S5. Structural homology modelling of PA14_45450 (PA1468) using Phyre2 (Kelley and Sternberg, 2009). Predicted structure of PA14_45450 (A and C) compared to the structure of the C-terminal receiver domain of the *E. coli* RcsC (B and D). The five characteristic α-helices are numbered from the N-terminal end of the polypeptide.

Table S1. Overview of protein-protein interactions tested by bacterial two-hybrid analysis. Domains of interest were cloned into the pUT18C or pKT25 vectors and cotransformed into *E. coli* DHM1 cells, which were spotted onto MacConkey agar. D stands for the receiver domain whereas H stands for the Hpt domain. Interactions were tested by colony colour (marked X), and positive interactions were confirmed by β-galactosidase assays (Miller Units). The 14 interactions of interest are shown in bold, while the other tests are controls. RocA1 and RocS1 were shown in previous studies to strongly interact and to control *cupB* and *cupC* genes expression (Kulasekara et al., 2005; Sivaneson et al., 2011).

**pUT18C / pKT25**	**E**	**RcsB (D)**	**PvrR (D)**	**PvrS (D)**
**E**	293	263	257	X
**RcsC(H)**	297	**608**	**233**	**X**
**RcsC(DH)**	274	**577**	**331**	
**HptA**	X	**X**	**X**	**X**
**HptB**	X	**X**	**X**	**X**
**HptC**	X	**X**	**X**	**X**
**TorR/TorS**	1709			
**RocA1 / RocS1**	1240			

Table S2. Supplementary strains and plasmids.

Strain/Plasmid	Relevant characteristics	Resistance*	Source
Escherichia coli			
TOP10 F'	Cloning strain: F' {*lacI*^{q} Tn10 (Tet^{r})} *mcrA* D(*mrr-hsd*RMS-*mcrBC*) φ80*lacZ*DM15 D*lac*χ74 *recA1 araD*139 D(*ara-leu*)7697 *galU galK rpsL* (Str^{r}) *endA1 nupG*	-	Invitrogen
OmniMAX	Cloning strain: F' {*proAB*$^{+}$ *lacI*^{q} *lacZ*DM15 Tn10(Tet^{r}) D (*ccdAB*)} *mcrA* D(*mrr-hsdRMS-mcrBC*) φ80(*lacZ*) DM15 D(*lacZYA-argF*) U169 *end*A1 *rec*A1 *supE*44 *thi*-1 *gyrA*96 *rel*A1 *ton*A *pan*D	Tc	Invitrogen
SM10 (λpir)	Donor strain, biparental mating: *leu, tonA, lacY, supE,* recA::RP4-2-Tc::Mu, lpir.	Km	Lab collection
1048	Helper strain for conjugation of plasmids by triparental mating: pRK2013, ColE1 ori tra+ mob+	Km	Lab collection
BL21	Expression strain for His-tagged RcsB	-	Lab collection
C41	Expression strain for His-tagged PvrS	-	Lab collection
Pseudomonas aeruginosa			
P_{BAD}-*rcsB-DZ*Δ*hptA*	P_{BAD}-*rcsB-DZ* with a clean deletion in *hptA*	-	This study
P_{BAD}-*rcsB-DZ*Δ*hptB*	P_{BAD}-*rcsB-DZ* with a clean deletion in *hptB*	-	This study
P_{BAD}-*rcsB-DZ*Δ*hptC*	P_{BAD}-*rcsB-DZ* with a clean deletion in *hptC*	-	This study
Plasmids			
pCR2.1-TA	Cloning vector for PCR products	Ap Km	Invitrogen
pCR-Blunt II-TOPO	Cloning vector for PCR products	Km	Invitrogen
pKNG101	Suicide vector for gene replacement in *P. aeruginosa*	Sm	Sarker & Cornelis (1997)
pKNG101-P_{BAD}-*rcsB*	Construct for inserting a P_{BAD} promoter in front of *rcsB*	Sm	This study
pKNG101D*rcsC*	Mutator for deletion of *rcsC*	Sm	Mikkelsen et al. (2009)
pKNG101D*rcsC*P_{BAD}	Mutator for deletion of *rcsC* in a P_{BAD}-*rcsB* strain background	Sm	This study
pKNG101D*pvrS*	Mutator for deletion of *pvrS*	Sm	Mikkelsen et al. (2009)
pKNG101D*pvrR*	Mutator for deletion of *pvrR*	Sm	Mikkelsen et al. (2009)

Strain/Plasmid	Relevant characteristics	Resistance*	Source
pKNG101D*hptA*	Mutator for deletion of *hptA*	Sm	This study
pKNG101D*hptB*	Mutator for deletion of *hptB*	Sm	Bordi et al. (2010)
pKNG101D*hptC*	Mutator for deletion of *hptC*	Sm	Isabelle Ventre
pBBR1-MCS-5-RcsB^{D71N}	*rcsB* with D71N mutation cloned into pBBR1-MCS-5	Gm	This study
pBBR1MCS-4-PvrR	*pvrR* cloned into pBBR1MCS-4	Ap	Mikkelsen et al. (2009)
pBBR1MCS-4-PvrR^{D57N}	PvrR-D57N cloned into pBBR1MCS-4	Ap	This study
pKT25-HptA	pKT25 carrying HptA	Km	Isabelle Ventre
pKT25-HptB	pKT25 carrying HptB	Km	Isabelle Ventre
pKT25-HptC	pKT25 carrying HptC	Km	Isabelle Ventre
pUT18c-PvrS-Rec	pUT18c carrying the Rec domain of PvrS	Ap	This study
pET28a-His-RcsB	*rcsB* cloned into pET28a, N-terminal His-tag	Km	This study
pET28a-His-RcsB^{D71N}	*rcsB* with D71N mutation cloned into pET28a, N-terminal His-tag	Km	This study
pET28a-His-PvrS	*pvrS* cloned into pET28a, N-terminal His-tag	Km	This study
pET28a-His-PvrS^{H463A}	*pvrS* with H463A mutation cloned into pET28a, N-terminal His-tag	Km	This study
pET28a-His-PvrS^{D862A}	*pvrS* with D862A mutation cloned into pET28a, N-terminal His-tag	Km	This study

Ap = ampicillin; Km = kanamycin; Sm = streptomycin; Tc = tetracycline; Gm = gentamicin.

Table S3. Complete list of genes that are modulated by *rcsB* overexpression (fold change ≥ 2; adjusted p-value ≤ 0.05).

PA number	Fold change	Corr. P-value	Gene Name	Alt. Gene Name	Product
**Up-regulated genes**					
PA14p00410	3.85	0.0357			putative dioxygenase
PA14p20060	2.12	0.0357			hypothetical protein
PA14p31150	2.14	0.0457			hypothetical protein
PA14p44090	2.43	0.0393			putative Fe-S-cluster oxidoreductase
PA14p45260	2.31	0.0424			hypothetical protein
PA14p50750	3.79	0.0378			hypothetical protein
PA14p54070	2.74	0.0383			hypothetical protein
PA14p59710	59.53	0.0357	cupD1	RL044	putative fimbrial protein precursor
PA14p59720	47.29	0.0449	cupD2	RL043	putative pili assembly chaperone
PA14p59750	6.73	0.0424	cupD4	RL041	fimbrial subunit
PA14p59760	8.42	0.0442	cupD5	RL040	putative pili assembly chaperone
PA14p59770	112.82	0.0393	rcsB	RL039	putative two component response regulator
PA14p59790	3.91	0.0357	pvrR	RL037	two component response regulator
PA14p59800	2.19	0.0442	pvrS	RL036	kinase sensor protein
PA0026	2.02	0.0445	plcB		phospholipase C, PlcB
PA0027	5.20	0.0491			hypothetical protein
PA0032	2.41	0.0402			probable transcriptional regulator
PA0033	10.38	0.0402	hptC		hptC
PA0034	8.59	0.0467			probable two-component response regulator
PA0062	2.97	0.0357			hypothetical protein
PA0125	2.01	0.0402			hypothetical protein
PA0267	6.81	0.0424			hypothetical protein (Rec domain)
PA0268	2.37	0.0402			probable transcriptional regulator
PA0309	3.44	0.0402			hypothetical protein
PA0338	2.53	0.0357			hypothetical protein
PA0361	3.25	0.0420			probable gamma-glutamyltranspeptidase precursor
PA0363	2.17	0.0433	coaD	kdtB	phosphopantetheine adenylyltransferase
PA0369	3.43	0.0402			hypothetical protein
PA0414	2.99	0.0437	chpB		probable methylesterase
PA0419	2.05	0.0442		yggJ	conserved hypothetical protein
PA0746	5.40	0.0499			probable acyl-CoA dehydrogenase
PA0747	2.64	0.0402			probable aldehyde dehydrogenase
PA0801	2.46	0.0402			hypothetical protein
PA0803	2.03	0.0449			hypothetical protein
PA0804	2.12	0.0467			probable oxidoreductase
PA0923	2.62	0.0484	dinB	dinP	DNA Polymerase IV, DinB
PA1020	2.14	0.0402			probable acyl-CoA dehydrogenase
PA1037	2.10	0.0424		yicG	conserved hypothetical protein
PA1059	2.56	0.0442	phaG	phaG	conserved hypothetical protein
PA1101	2.43	0.0450	fliF		Flagella M-ring outer membrane protein precursor
PA1104	2.94	0.0467	fliI		flagellum-specific ATP synthase FliI
PA1109	2.64	0.0476			probable transcriptional regulator
PA1165	2.70	0.0191	pcpS		PcpS
PA1173	2.09	0.0445	napB		cytochrome c-type protein NapB precursor
PA1228	3.77	0.0402			hypothetical protein
PA1234	2.76	0.0467			hypothetical protein

PA number	Fold change	Corr. P-value	Gene Name	Alt. Gene Name	Product
PA1245	2.19	0.0498	aprX	aprX	AprX
PA1465	3.92	0.0499			hypothetical protein
PA1468	9.89	0.0357			hypothetical protein
PA1470	2.84	0.0357			probable short-chain dehydrogenase
PA1476	2.31	0.0402	ccmB	helB	heme exporter protein CcmB
PA1484	2.39	0.0467			probable transcriptional regulator
PA1571	4.46	0.0420			hypothetical protein
PA1575	2.50	0.0402			hypothetical protein
PA1638	2.06	0.0466		yneH	conserved hypothetical protein
PA1641	2.07	0.0495			hypothetical protein
PA1642	2.29	0.0467	selD		selenophosphate synthetase
PA1664	9.43	0.0393		orfX	hypothetical protein
PA1668	2.94	0.0434		dotU2	hypothetical protein
PA1669	2.70	0.0424		icmF2	hypothetical protein
PA1755	2.46	0.0484			hypothetical protein
PA1823	3.05	0.0495		yjaD	conserved hypothetical protein
PA1870	2.28	0.0449			hypothetical protein
PA1871	2.47	0.0357	lasA		LasA protease precursor
PA1890	2.82	0.0357			probable glutathione S-transferase
PA1959	2.48	0.0498	bacA		bacitracin resistance protein
PA1960	2.71	0.0478			hypothetical protein
PA1968	2.30	0.0467			hypothetical protein
PA1970	2.14	0.0495			hypothetical protein
PA2005	2.93	0.0479			probable transcriptional regulator
PA2010	2.02	0.0449			probable transcriptional regulator
PA2020	2.55	0.0442	amrR	mexZ	probable transcriptional regulator
PA2063	2.78	0.0442			hypothetical protein
PA2066	2.12	0.0486			hypothetical protein
PA2075	4.11	0.0424			hypothetical protein
PA2111	6.47	0.0468			hypothetical protein
PA2116	3.17	0.0467			conserved hypothetical protein
PA2253	2.45	0.0499	ansA		L-asparaginase I
PA2258	3.66	0.0378	ptxR		transcriptional regulator PtxR
PA2274	3.29	0.0442			hypothetical protein
PA2279	2.35	0.0465	arsC		ArsC protein
PA2332	2.93	0.0424			probable transcriptional regulator
PA2344	2.41	0.0424	mtlZ	frk	fructokinase
PA2346	2.19	0.0473			conserved hypothetical protein
PA2442	2.64	0.0420	gcvT2		glycine cleavage system protein T2
PA2476	2.25	0.0481	dsbG		thiol:disulfide interchange protein DsbG
PA2489	2.73	0.0449			probable transcriptional regulator
PA2495	3.00	0.0402	oprN		Multidrug efflux outer membrane protein OprN precursor
PA2502	2.38	0.0357			hypothetical protein
PA2506	3.04	0.0449			hypothetical protein
PA2543	2.30	0.0445		ytfM	conserved hypothetical protein
PA2553	6.02	0.0481			probable acyl-CoA thiolase
PA2554	5.95	0.0449			probable short-chain dehydrogenase
PA2555	3.28	0.0424			probable AMP-binding enzyme
PA2557	4.78	0.0484			probable AMP-binding enzyme
PA2605	7.59	0.0393	dsrE	yheN	conserved hypothetical protein
PA2692	3.31	0.0424			probable transcriptional regulator

PA number	Fold change	Corr. P-value	Gene Name	Alt. Gene Name	Product
PA2727	2.03	0.0357			hypothetical protein
PA2728	2.13	0.0424			hypothetical protein
PA2780	2.79	0.0402			hypothetical protein
PA2832	3.43	0.0357	tpm		thiopurine methyltransferase
PA2883	2.67	0.0407			hypothetical protein
PA2890	2.43	0.0357	atuE		putative isohexenylglutaconyl-CoA hydratase
PA2894	3.73	0.0424			hypothetical protein
PA2895	2.73	0.0442			hypothetical protein
PA2896	2.03	0.0445			probable sigma-70 factor, ECF subfamily
PA2908	3.00	0.0402	cbiD		cobalamin biosynthetic protein CbiD
PA2923	2.10	0.0357	hisJ		periplasmic histidine-binding protein HisJ
PA2930	2.20	0.0442			probable transcriptional regulator
PA2942	2.69	0.0393			probable magnesium chelatase
PA2981	3.27	0.0402	lpxK		tetraacyldisaccharide 4*-kinase
PA3069	2.46	0.0420			hypothetical protein
PA3074	2.61	0.0378			hypothetical protein
PA3091	3.39	0.0424			hypothetical protein
PA3095	2.80	0.0442	xcpZ		general secretion pathway protein M
PA3110	2.95	0.0357			hypothetical protein
PA3113	2.19	0.0495	trpF		N-(5'phosphoribosyl)anthranilate (PRA) isomerase
PA3125	2.14	0.0468			hypothetical protein
PA3129	2.53	0.0402		yohI	conserved hypothetical protein
PA3178	3.32	0.0495			hypothetical protein
PA3179	4.31	0.0442		yciL	conserved hypothetical protein
PA3209	3.44	0.0495		ykgJ	conserved hypothetical protein
PA3248	2.34	0.0465			hypothetical protein
PA3272	2.09	0.0424		lhr	probable ATP-dependent DNA helicase
PA3304	3.03	0.0424			conserved hypothetical protein
PA3316	2.62	0.0402			probable permease of ABC transporter
PA3328	3.78	0.0445			probable FAD-dependent monooxygenase
PA3330	2.73	0.0442			probable short chain dehydrogenase
PA3458	3.21	0.0442			probable transcriptional regulator
PA3459	3.43	0.0479		asnB	probable glutamine amidotransferase
PA3493	2.83	0.0357		rnfG	conserved hypothetical protein
PA3526	2.93	0.0424		motY	probable outer membrane protein precursor
PA3555	3.41	0.0449	arnD	amrJ	ArnD
PA3556	2.73	0.0467	arnT	pqaB	inner membrane L-Ara4N transferase ArnT
PA3572	3.71	0.0357			hypothetical protein
PA3669	2.00	0.0499			hypothetical protein
PA3685	3.16	0.0495			conserved hypothetical protein
PA3714	4.54	0.0471			probable two-component response regulator
PA3856	2.36	0.0491			hypothetical protein
PA3952	3.09	0.0449			hypothetical protein
PA3969	2.31	0.0495			conserved hypothetical protein
PA3985	2.16	0.0357			conserved hypothetical protein
PA4019	2.37	0.0449			probable aromatic acid decarboxylase
PA4027	2.15	0.0402			hypothetical protein
PA4128	2.86	0.0442		hpaI	conserved hypothetical protein
PA4201	2.62	0.0442	ddlA		D-alanine-D-alanine ligase A
PA4208	8.62	0.0433	opmD		probable outer membrane protein precursor
PA4226	3.45	0.0437	pchE		dihydroaeruginoic acid synthetase

PA number	Fold change	Corr. P-value	Gene Name	Alt. Gene Name	Product
PA4322	2.19	0.0442			conserved hypothetical protein
PA4350	3.01	0.0442	olsB		OlsB
PA4475	2.31	0.0442			conserved hypothetical protein
PA4706	2.36	0.0424		phuV	probable ATP-binding component of ABC transporter
PA4780	2.30	0.0424			conserved hypothetical protein
PA4899	3.11	0.0424			probable aldehyde dehydrogenase
PA4906	2.54	0.0357	vanR		probable transcriptional regulator
PA4946	2.21	0.0393	mutL		DNA mismatch repair protein MutL
PA4966	2.96	0.0500			hypothetical protein
PA4988	2.80	0.0424	waaA	kdtA	3-deoxy-D-manno-octulosonic-acid (KDO) transferase
PA5006	2.01	0.0442			hypothetical protein
PA5116	2.66	0.0440			probable transcriptional regulator
PA5184	2.50	0.0495			hypothetical protein
PA5189	2.32	0.0357			probable transcriptional regulator
PA5202	2.34	0.0442			hypothetical protein
PA5221	3.01	0.0393		visC	probable FAD-dependent monooxygenase
PA5228	2.10	0.0476		ygfA	conserved hypothetical protein
PA5229	2.02	0.0495			conserved hypothetical protein
PA5258	2.08	0.0442		hemX	hypothetical protein
PA5261	2.32	0.0448	algR		alginate biosynthesis regulatory protein AlgR
PA5308	2.07	0.0482	lrp	dadR	leucine-responsive regulatory protein
PA5467	3.26	0.0422			hypothetical protein
PA5526	2.09	0.0407			hypothetical protein
PA5533	2.43	0.0357			hypothetical protein
**Down-regulated genes**					
PA14p01220	3.16	0.0424			Hypothetical protein
PA14p10050	2.43	0.0445			hypothetical protein
PA14p10960	4.09	0.0357			ferridoxin oxidoreductase subunit
PA14p30960	2.35	0.0442	TraG		conjugal transfer coupling protein TraG
PA14p31110	2.85	0.0442			putative replication initiator and transcriptional repressor protein
PA14p31250	2.94	0.0402			hypothetical protein
PA14p35940	2.14	0.0442			acyl-CoA synthetase
PA14p36400	4.00	0.0388			hypothetical protein
PA14p36860	5.50	0.0475			hypothetical protein
PA14p39670	2.99	0.0477			hypothetical protein
PA14p39700	5.81	0.0442			hypothetical protein
PA14p48230	4.49	0.0449			hypothetical protein
PA14p53100	2.36	0.0442			hypothetical protein
PA14p55400	2.74	0.0357			hypothetical protein
PA14p59030	3.47	0.0495		RL105	hypothetical protein
PA14p59240	2.06	0.0498	pilL2	RL086	type IV B pilus protein
PA14p59280	3.44	0.0393	pilP2	RL083	type IV B pilus protein
PA14p59410	2.91	0.0420		RL073	hypothetical Protein
PA14p59430	2.99	0.0445		RL072	hypothetical protein
PA14p59520	2.05	0.0402		RL065	hypothetical protein
PA14p59870	2.02	0.0445		RL029	hypothetical protein
PA14p64260	3.49	0.0393			hypothetical protein
PA14p64280	3.86	0.0448			probable permease of ABC transporter
PA14p71400	4.07	0.0445			hypothetical protein
PA0044	2.94	0.0357	exoT		exoenzyme T

PA number	Fold change	Corr. P-value	Gene Name	Alt. Gene Name	Product
PA0051	3.59	0.0442	phzH		potential phenazine-modifying enzyme
PA0091	3.55	0.0357	vgrG1		VgrG1
PA0096	2.12	0.0499			hypothetical protein
PA0098	2.18	0.0475			hypothetical protein
PA0107	4.09	0.0402			conserved hypothetical protein
PA0108	4.45	0.0357	coIII		cytochrome c oxidase, subunit III
PA0109	2.35	0.0449			hypothetical protein
PA0111	2.27	0.0357			hypothetical protein
PA0118	2.75	0.0449			hypothetical protein
PA0137	3.15	0.0402			probable permease of ABC transporter
PA0138	3.49	0.0445			probable permease of ABC transporter
PA0142	2.04	0.0442			hypothetical protein
PA0152	2.40	0.0465	pcaQ		transcriptional regulator PcaQ
PA0154	3.16	0.0357	pcaG		protocatechuate 3,4-dioxygenase, alpha subunit
PA0164	2.02	0.0498			probable gamma-glutamyltranspeptidase
PA0166	2.00	0.0393			probable transporter
PA0171	2.28	0.0445			hypothetical protein
PA0173	2.75	0.0388			probable methylesterase
PA0182	2.04	0.0449		yjgI	probable short-chain dehydrogenase
PA0183	2.63	0.0402	atsA		arylsulfatase
PA0192	2.95	0.0424			probable TonB-dependent receptor
PA0193	2.24	0.0484			hypothetical protein
PA0194	2.65	0.0449			hypothetical protein
PA0198	2.61	0.0357	exbB1		transport protein ExbB
PA0199	2.64	0.0357	exbD1		transport protein ExbD
PA0215	3.91	0.0357		madL	malonate transporter MadL
PA0216	3.38	0.0486		madM	malonate transporter MadM
PA0222	3.26	0.0397			hypothetical protein
PA0241	3.52	0.0357			probable major facilitator superfamily (MFS) transporter
PA0242	2.72	0.0357			hypothetical protein
PA0256	2.30	0.0424			hypothetical protein
PA0287	2.25	0.0456	gpuP	speB1	3-guanidinopropionate transport protein
PA0289	2.30	0.0442	gpuR		transcriptional activator GpuR
PA0434	2.14	0.0448			hypothetical protein
PA0435	5.12	0.0357			hypothetical protein
PA0439	4.57	0.0393		dypB;yeiA	probable oxidoreductase
PA0443	4.13	0.0357			probable transporter
PA0459	2.24	0.0357		clpC	probable ClpA/B protease ATP binding subunit
PA0513	3.48	0.0402		nirG	probable transcriptional regulator
PA0515	2.75	0.0495		nirD	probable transcriptional regulator
PA0516	3.32	0.0442	nirF		heme d1 biosynthesis protein NirF
PA0519	3.55	0.0357	nirS		nitrite reductase precursor
PA0524	3.07	0.0442	norB		nitric-oxide reductase subunit B
PA0531	3.07	0.0393			probable glutamine amidotransferase
PA0545	2.13	0.0491			hypothetical protein
PA0681	2.47	0.0445		hxcT	HxcT pseudopilin
PA0687	2.13	0.0402		hxcS	probable type II secretion system protein
PA0692	2.43	0.0383			hypothetical protein
PA0718	2.77	0.0402			hypothetical protein of bacteriophage Pf1
PA0819	2.32	0.0481			hypothetical protein

PA number	Fold change	Corr. P-value	Gene Name	Alt. Gene Name	Product
PA0844	2.45	0.0357	plcH	phlC;plcS	hemolytic phospholipase C precursor
PA0864	2.20	0.0388			probable transcriptional regulator
PA0875	2.55	0.0442			conserved hypothetical protein
PA1028	2.25	0.0488			probable oxidoreductase
PA1134	2.18	0.0442			hypothetical protein
PA1137	2.95	0.0442			probable oxidoreductase
PA1174	2.11	0.0357	napA		periplasmic nitrate reductase protein NapA
PA1195	2.03	0.0357			hypothetical protein
PA1230	4.08	0.0424			hypothetical protein
PA1236	3.72	0.0449			probable major facilitator superfamily (MFS) transporter
PA1257	2.80	0.0467			amino acid ABC transporter membrane protein
PA1259	2.14	0.0423			hypothetical protein
PA1404	2.13	0.0442			hypothetical protein
PA1408	2.37	0.0449			hypothetical protein
PA1485	3.54	0.0402			probable amino acid permease
PA1499	2.56	0.0424			conserved hypothetical protein
PA1519	2.15	0.0442			probable transporter
PA1692	2.03	0.0424		pscS	probable translocation protein in type III secretion
PA1706	2.23	0.0428	pcrV		type III secretion protein PcrV
PA1708	3.28	0.0495	popB	pepB	translocator protein PopB
PA1719	2.16	0.0437	pscF		type III export protein PscF
PA1724	2.11	0.0420	pscK		type III export protein PscK
PA1743	2.12	0.0437			hypothetical protein
PA1779	2.17	0.0357		nasC;nasA	assimilatory nitrate reductase
PA1855	5.38	0.0449			hypothetical protein
PA1917	3.39	0.0402			hypothetical protein
PA1922	2.20	0.0442		feuA;cirA	probable TonB-dependent receptor
PA1924	2.10	0.0445			hypothetical protein
PA1975	2.00	0.0442			hypothetical protein
PA1984	3.12	0.0460	exaC	exaC1	NAD+ dependent aldehyde dehydrogenase ExaC
PA2057	3.09	0.0498			hypothetical protein
PA2086	3.21	0.0442			probable epoxide hydrolase
PA2140	3.85	0.0393			probable metallothionein
PA2163	2.04	0.0442			hypothetical protein
PA2168	2.67	0.0424			hypothetical protein
PA2178	3.36	0.0357			hypothetical protein
PA2189	3.94	0.0424			hypothetical protein
PA2191	3.08	0.0439	exoY		adenylate cyclase ExoY
PA2213	2.34	0.0442	opdG	opdG	probable porin
PA2214	3.26	0.0437	yfaV		probable major facilitator superfamily (MFS) transporter
PA2259	2.61	0.0495	ptxS		transcriptional regulator PtxS
PA2283	2.24	0.0357			hypothetical protein
PA2400	2.10	0.0449	pvdJ		PvdJ
PA2418	2.24	0.0449			hypothetical protein
PA2420	2.36	0.0357		opdJ	probable porin
PA2474	2.93	0.0393			hypothetical protein
PA2562	2.21	0.0449			hypothetical protein
PA2673	2.39	0.0357		hplV	probable type II secretion system protein
PA2746	2.68	0.0445			hypothetical protein

PA number	Fold change	Corr. P-value	Gene Name	Alt. Gene Name	Product
PA2845	6.72	0.0445			hypothetical protein
PA2847	2.88	0.0357			conserved hypothetical protein
PA2898	2.15	0.0484			hypothetical protein
PA2910	2.22	0.0462		yebN	conserved hypothetical protein
PA3289	2.34	0.0484			hypothetical protein
PA3311	3.49	0.0495			conserved hypothetical protein
PA3383	2.79	0.0466		phnD	binding protein component of ABC phosphonate transporter
PA3408	2.56	0.0357	hasR		Heme uptake outer membrane receptor HasR precursor
PA3451	3.19	0.0393			hypothetical protein
PA3465	2.43	0.0442		yfiS	conserved hypothetical protein
PA3516	2.03	0.0499			probable lyase
PA3534	2.07	0.0486			probable oxidoreductase
PA3541	3.02	0.0420	alg8		alginate biosynthesis protein Alg8
PA3542	2.40	0.0442	alg44		alginate biosynthesis protein Alg44
PA3589	2.41	0.0357	paaJ		probable acyl-CoA thiolase
PA3590	2.05	0.0467			probable hydroxyacyl-CoA dehydrogenase
PA3592	3.96	0.0420		baiF	conserved hypothetical protein
PA3597	3.12	0.0442	ycjJ		probable amino acid permease
PA3709	2.26	0.0495			probable major facilitator superfamily (MFS) transporter
PA3775	3.70	0.0402			hypothetical protein
PA3842	2.23	0.0486	spcS		specific Pseudomonas chaperone for ExoS, SpcS
PA3843	3.42	0.0449			hypothetical protein
PA3873	2.76	0.0465	narJ		respiratory nitrate reductase delta chain
PA3875	3.06	0.0424	narG		respiratory nitrate reductase alpha chain
PA3877	2.76	0.0420	narK1		nitrite extrusion protein 1
PA3892	2.47	0.0463			conserved hypothetical protein
PA3893	2.16	0.0481			conserved hypothetical protein
PA3909	2.40	0.0442	eddB		Extracelullar DNA degradation protein, EddB
PA3912	2.54	0.0449		yhbV	conserved hypothetical protein
PA3946	2.01	0.0424	rocS1		Two-component sensor RocS1
PA3964	3.31	0.0437			hypothetical protein
PA3994	2.30	0.0402			probable epoxide hydrolase
PA4071	5.34	0.0378			hypothetical protein
PA4072	3.27	0.0402			probable amino acid permease
PA4082	3.73	0.0424	cupB5		adhesive protein CupB5
PA4084	2.14	0.0402	cupB3	htrE	usher CupB3
PA4092	2.64	0.0445	hpaC		4-hydroxyphenylacetate 3-monooxygenase small chain
PA4096	3.82	0.0407	phlE	phlE	probable major facilitator superfamily (MFS) transporter
PA4097	2.47	0.0455	ydjL	ydjL	probable alcohol dehydrogenase (Zn-dependent)
PA4099	4.70	0.0357			hypothetical protein
PA4123	2.67	0.0455	hpcC	hpaE	5-carboxy-2-hydroxymuconate semialdehyde dehydrogenase
PA4127	2.26	0.0449	hpcG	hpaH	2-oxo-hept-3-ene-1,7-dioate hydratase
PA4140	2.55	0.0357			hypothetical protein
PA4150	4.07	0.0424		acoA	probable dehydrogenase E1 component
PA4151	3.10	0.0393	acoB		acetoin catabolism protein AcoB
PA4540	2.18	0.0460		lepB	hypothetical protein

PA number	Fold change	Corr. P-value	Gene Name	Alt. Gene Name	Product
PA4610	3.29	0.0420			hypothetical protein
PA4625	2.21	0.0442	cdrA		cyclic diguanylate-regulated TPS partner A, CdrA
PA4649	2.54	0.0357	cupE2		Pilin subunit CupE3
PA4652	2.53	0.0357	cupE5		Fimbrial usher protein CupE5
PA4653	2.51	0.0455	cupE6		Adhesin-like protein CupE6
PA4799	2.03	0.0445			hypothetical protein
PA4804	3.40	0.0456			hypothetical protein
PA4814	2.82	0.0499	fadH2		2,4-dienoyl-CoA reductase FadH2
PA4820	2.34	0.0467			hypothetical protein
PA4828	2.88	0.0479			conserved hypothetical protein
PA4834	3.18	0.0402			hypothetical protein
PA4860	6.89	0.0407			probable permease of ABC transporter
PA4882	5.31	0.0357			hypothetical protein
PA4891	2.51	0.0410	ureE		urease accessory protein UreE
PA4908	2.60	0.0442			hypothetical protein
PA4912	2.56	0.0402			branched chain amino acid ABC transporter membrane protein
PA4981	2.69	0.0357			probable amino acid permease
PA4989	2.46	0.0475			probable transcriptional regulator
PA4993	2.14	0.0484			hypothetical protein
PA5102	3.01	0.0440			hypothetical protein
PA5353	2.35	0.0442	glcF		glycolate oxidase subunit GlcF
PA5379	3.17	0.0407	sdaB		L-serine dehydratase
PA5387	3.46	0.0402	cdhC		CdhC, Carnitine dehydrogenase-related gene C
PA5393	3.12	0.0450			conserved hypothetical protein
PA5396	2.36	0.0424			hypothetical protein
PA5410	2.71	0.0424	gbcA		GbcA
PA5416	2.64	0.0491	soxB		sarcosine oxidase beta subunit
PA5418	2.11	0.0378	soxA		sarcosine oxidase alpha subunit
PA5419	2.59	0.0464	soxG		sarcosine oxidase gamma subunit
PA5512	2.16	0.0498	mifS (dctB in PA14)		MifS
PA5532	2.49	0.0442			hypothetical protein
PA5534	2.54	0.0491			hypothetical protein
PA5539	3.22	0.0388			hypothetical protein
PA5541	2.86	0.0393	pyrQ	pyrC2	dihydroorotase
PA5544	2.22	0.0445			conserved hypothetical protein

Table S4. Oligonucleotides used in this study.

Construct / Oligo No.	Oligo name	Sequence (5' -> 3')	Description	Source
**MUTATORS**				
**pKNG101-P_{BAD}-*rcsB*: Introducing the P_{BAD} promoter in front of *rcsB* on the chromosome**				
OAL266	P_{BAD} F	AA CATATG CGTCAATTGTCTGATTCGTTACCAAT	*araC*-PBAD, *Nde*I site	This study
OAL267	P_{BAD} R	AA TC GCTAGC CCAAAAAAACGG	*araC*-PBAD, *Nhe*I site	This study
OAL262	P_{BAD} Up F	AAA GTCGAC CGTCAACATGCCGAACATGAC	*rcsC* fragment, *Sal*I site	This study
OAL263	P_{BAD} Up R	TCG GCTAGC AAA CATATG CATCAGCCCGTCTTTCTCACAA	*rcsC* fragment, *Nhe*I and *Nde*I sites	This study
OAL264	P_{BAD} Down F	ATG CATATG TTT GCTAGC CGAGGAGGATCTAGA ATGCGTCCGGGGTCAATAGT	*rcsB* fragment, *Nde*I and *Nhe*I sites	This study
OAL265	P_{BAD} Down R	AA GGGCCC TCGAACTCCTTCATCGACAAGC	*rcsB* fragment, ApaI site	This study
**pKNG101D*rcsC*P_{BAD}: Deletion of *rcsC* in a P_{BAD}-*rcsB* background**				
OGBD5	drcsC1	AA GGGCCC TCGAACTCCTTCATCGACAAGC	*rcsC* upstream fragment in P_{BAD}-*rcsB* background	Mikkelsen et al. (2009)
OAL851	drcsC2	TCAGCCCGT AGACTTCACGGGATGCGG	*rcsC* upstream fragment in P_{BAD}-*rcsB* background	This study
OAL852	drcsC3	GTGAAGTCT ACGGGCTGATG CATATG CG	*rcsC* downstrream fragment in P_{BAD}-*rcsB* background	This study
OAL853	drcsC4	GAGCTGCTGGCGATAAATCT	*rcsC* downstrream fragment in P_{BAD}-*rcsB* background	This study
**pKNG101D*hptA*: Deletion of *hptA***				
OAL473	dhptA1	TAGAACCCTCGCTGCTCGAT	*hptA* upstream fragment	This study
OAL474	dhptA2	CTAGAGCCT CGCTTTCAT ACAGACTCCATCGATATG	*hptA* upstream fragment	This study
OAL475	dhptA3	ATGAAAGCG AGGCTCTAG AGATCGGGCTGG	*hptA* downstream fragment	This study
OAL476	dhptA4	AAATTGATGGTGCCGTCTGC	*hptA* downstream fragment	This study
**pKNG101D*hptC*: Deletion of *hptC***				
	CupA	GCTCTAGAAAAGAAGGTTCGCATGAGTAAGGTG	*hptC* upstream fragment, *Xba*I	Isabelle Ventre
	CupB	CGTGAATTCCATAGCGTTGCGGGCCTTCGTCCAG	*hptC* upstream fragment, *Eco*RI	Isabelle Ventre
	CloA	ATGGAATTCACGTTCGCATGGGATCCTCAGATCA	*hptC* downstream fragment, *Eco*RI	Isabelle Ventre
	CloB	GGACTAGTACGGTTTCGTCGACTGGCTACAGGC	*hptC* downstream fragment, *Spe*I	Isabelle Ventre
**OVEREXPRESSION CONSTRUCTS**				
**pBBR1MCS-4-*rcsC***				
OCG39	rcsC F	ACGCAGTATCCCCATTATCG	*rcsC* upstream	Genevieve Ball
OCG40	rcsC R	TCGGCCAGTACTATCTTGAGC	*rcsC* downstream	Genevieve Ball
**pBBR1MCS-4-*pvrS***				
OCG33	pvrS F	GGAGGCCGTCACTGTAGATT	*pvrS* upstream	Genevieve Ball
OCG34	pvrS R	CCGATAGGATTTCCAGCTCA	*pvrS* downstream	Genevieve Ball
**BACTERIAL TWO HYBRID CONSTRUCTS**				
OAL181	BTHC-up	GCTCTAGAG AGTGAGTTCGCTCGCG	Hpt domain of RcsC for T25 fusion	This study
OAL182	BTHC-down	CGCGGTACCTCAGCCCGTCTTTCTCAC	Hpt domain of RcsC for T25 fusion	This study
OAL577	B2H-rcsC F	GC TCTAGAG CAAACGCCGGCAAATG	Receiver and Hpt domains of RcsC for T25 fusion	This study
OAL183	BTHS-up	GCT CTA GAG GAG CGG ATC GCG ATG	Receiver domain of PvrS for T18 fusion	This study
OAL185	BTHS-down	CGG GGT ACC TCA TCG ATC CAC CTT GAG AAT	Receiver domain of PvrS for T18 fusion	This study
OAL186	BTHB-up	GCT CTA GAG ATG CGT CCG GGG TCA	Receiver domain of RcsB for T18 fusion	This study
OAL187	BTHB-down	A GGT ACC CTA GCGGCTTTCCACTTCGT	Receiver domain of RcsB for T18 fusion	This study
OAL188	BTHR-up	CGT CTA GAG ATG AGC TGG AAA TCC TAT CG	Receiver domain of PvrR for T18 fusion	This study
OAL189	BTHR-down	A GGT ACC TTA CCCATGCGGAGTCTCG	Receiver domain of PvrR for T18 fusion	This study
	UHptA	AAAAGCTGCAGGAGTCTGTATGAAAGAGCTTGGTTCGGAA	HptA (*Pst*I) for T25 fusion	Isabelle Ventre

Construct / Oligo No.	Oligo name	Sequence (5' -> 3')	Description	Source
	LHptA	CGGGGTACCATTCTGAAACCTCTATCTGGTCCGATCTCTA	HptA (*Kpn*I) for T25 fusion	Isabelle Ventre
	UHptB	GCTCTAGAGCGAATGTCCGCGCCGCATCTCGATGATCGTG	HptB (*Xba*I) for T25 fusion	Isabelle Ventre
	LHptB	GGGCCGGTACCTTGTCGCCGGAAAGGACGAAAACCTCAGC	HptB (*Kpn*I) for T25 fusion	Isabelle Ventre
	UHptC	GCTCTAGAGTCGCCCGCCGCCACGAGCTCATCTGAGGA	HptC (*Xba*I) for T25 fusion	Isabelle Ventre
	LHptC	CGGGGTACCAGATCCTGGACGAAGGCCCGCAACGATATGA	HptC (*Kpn*I) for T25 fusion	Isabelle Ventre
**Site directed mutagenesis**				
OAL390	rcsBg211a F	GCATATCGCCATTACCAACTACAGCATGCCCGG	RcsB-D71N	This study
OAL391	rcsBg211a R	CCGGGCATGCTGTAGTTGGTAATGGCGATATGC	RcsB-D71N	This study
OAL575	pvrRt171a F	CTGATCCTCAGCGAACTGATGATGCCGGG	PvrR-D57N	This study
OAL576	pvrRt171a R	CCCGGCATCATCAGTTCGCTGAGGATCAG	PvrR-D57N	This study
OAL703	rcsC-a1516gF	AGCCATGAAATCCGTGCTCCCCTGTACGGTG	RcsC-T506A	This study
OAL704	rcsC-a1516gR	CACCGTACAGGGGAGCACGGATTTCATGGCT	RcsC-T506A	This study
OAL942	c1504g_a1505c F	CTGGCCCGCATGAGCGCTGAAATCCGTGCTCC	RcsC-H502A-T506A	This study
OAL943	c1504g_a1505c R	GGAGCACGGATTTCAGCGCTCATGCGGGCCAG	RcsC-H502A-T506A	This study
OAL940	c3085g_a3086c F	GCAGCAGGACCTGGCTCGCATGGCCGGC	RcsC-H1029A	This study
OAL941	c3085g_a3086c R	GCCGGCCATGCGAGCCAGGTCCTGCTGC	RcsC-H1029A	This study
OAL705	pvrS-c1387g_a1388cF	CTCGCCACCATGAGCGCTGAAATCCGCACACC	PvrS-H424A	This study
OAL706	pvrS-c1387g_a1388cR	GGTGTGCGGATTTCAGCGCTCATGGTGGCGAG	PvrS-H424A	This study
OAL893	pvrS-a2585cF	GACGTGGTGCTCACCGCTATCAACATGCCGAAC	PvrS-D862A	This study
OAL894	pvrS-a2585cR	GTTCGGCATGTTGATAGCGGTGAGCACCACGTC	PvrS-D862A	This study
OAL930	g452t_a453t_a454c F	GCGAGAAACCCACTTTCGGTCCTTTAATTCACGTCCTAAATA CGCC	Mutated P*cupD* for *cupD1.1-lacZ*	This study
OAL931	g452t_a453t_a454c R	GGCGTATTTAGGACGTGAATTAAAGGACCGAAAGTGGGTTTC TCGC	Mutated P*cupD* for *cupD1.1-lacZ*	This study
OAL932	g372t_a373t_a374c F	GCCTCGCTATGAGGCCCGTTCATGTCTATTGGAGCAGGG	Mutated P*cupD* for *cupD1.2-lacZ*	This study
OAL933	g372t_a373t_a374c R	CCCTGCTCCAATAGACATGAACGGGCCTCATAGCGAGGC	Mutated P*cupD* for *cupD1.2-lacZ*	This study
**His-tagged constructs**				
OAL479	rcsB F	CCCATATGCGTCCGGGGTCAATAG	*rcsB* (*Nde*I) for sub-cloning into pET28a	This study
OCG38	rcsB R	GCTTCAGCTGGATCGATGAC	*rcsB* downstream	Mikkelsen et al. (2009)
OAL1639	pvrS F	AAAGCTAGC ATGAAGTTGAAGAATTTCTT	*pvrS* (*Nhe*I), used with OCG34	This study

Supplementary References

Bordi, C., Lamy, M. C., Ventre I, Termine, E., Hachani, A., Fillet, S., Roche, B., Bleves, S., Méjean, V., Lazdunski, A. & A. Filloux, (2010) Regulatory RNAs and the HptB/RetS signalling pathways fine-tune *Pseudomonas aeruginosa* pathogenesis. *Mol Microbiol* **76:** 1427-1443.

Hachani, A., Lossi, N. S., Hamilton, A., Jones, C., Bleves, S., Albesa-Jové, D. & A. Filloux, (2011) Type VI secretion system in *Pseudomonas aeruginosa*: secretion and multimerization of VgrG proteins. *J Biol Chem* **286:** 12317-12327.

Sarker, M. R. & G. R. Cornelis, (1997) An improved version of suicide vector pKNG101 for gene replacement in gram-negative bacteria. *Mol Microbiol* **23:** 410-411.
